# “Toolbox” for the Processing of Functional Polymer Composites

**DOI:** 10.1007/s40820-021-00774-5

**Published:** 2021-12-16

**Authors:** Yun Wei, Hongju Zhou, Hua Deng, Wenjing Ji, Ke Tian, Zhuyu Ma, Kaiyi Zhang, Qiang Fu

**Affiliations:** College of Polymer Science and Engineering, State Key Laboratory of Polymer Materials Engineering, Sichuan University, Chengdu, 610065 People’s Republic of China

**Keywords:** Toolbox, Functional polymer composites, Processing strategy, Morphology control

## Abstract

- The processing methods of functional polymer composites (FPCs) are systematically summarized in “Toolbox”.
- The relationship of processing method-structure-property is discussed and the selection and combination of tools in processing among different FPCs are analyzed.
- A promising prospect is provided regarding the design principle for high performance FPCs for further investigation.

**Abstract:**

Functional polymer composites (FPCs) have attracted increasing attention in recent decades due to their great potential in delivering a wide range of functionalities. These functionalities are largely determined by functional fillers and their network morphology in polymer matrix. In recent years, a large number of studies on morphology control and interfacial modification have been reported, where numerous preparation methods and exciting performance of FPCs have been reported. Despite the fact that these FPCs have many similarities because they are all consisting of functional inorganic fillers and polymer matrices, review on the overall progress of FPCs is still missing, and especially the overall processing strategy for these composites is urgently needed. Herein, a “Toolbox” for the processing of FPCs is proposed to summarize and analyze the overall processing strategies and corresponding morphology evolution for FPCs. From this perspective, the morphological control methods already utilized for various FPCs are systematically reviewed, so that guidelines or even predictions on the processing strategies of various FPCs as well as multi-functional polymer composites could be given. This review should be able to provide interesting insights for the field of FPCs and boost future intelligent design of various FPCs.
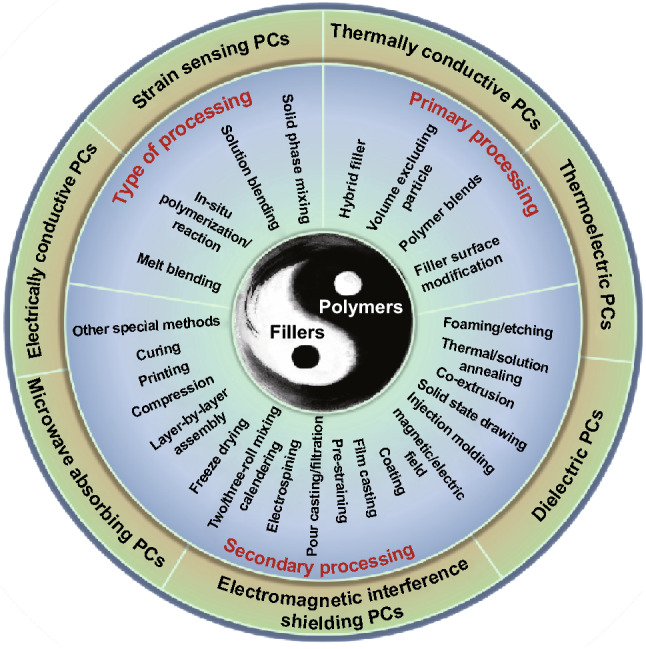

**Supplementary Information:**

The online version contains supplementary material available at 10.1007/s40820-021-00774-5.

## Introduction

**Fig. 1:**
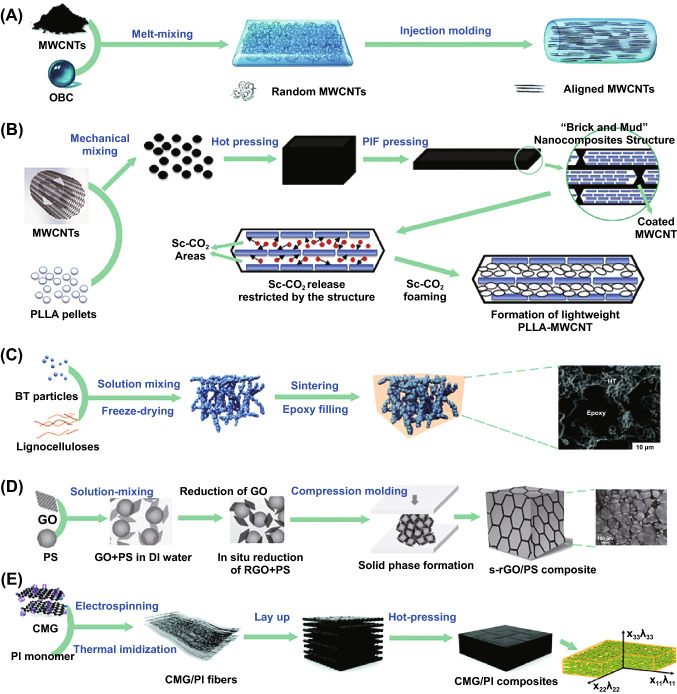
**A** Schematic for the preparation and morphology of ethylene-α-octene block copolymer (OBC)/multi-walled carbon nanotubes (MWCNTs) via melt blending and injection molding. Reprinted with permission from Ref. [17]. **B** Schematics of using pressure-induced flow (PIF) and supercritical carbon dioxide (Sc-CO_2_) to prepare poly(l-lactide) (PLLA)/MWCNTs foams. Reprinted with permission from Ref. [18]. **C** Schematic illustration of the epoxy/BaTiO_3_ (BT) composites fabrication process through solution blending BT with lignocellulose, freeze drying, sintering and filling with epoxy in sequence. Reprinted with permission from Ref. [19]. **D** Schematic of the fabrication and structure reduced graphene oxide/polystyrene (rGO/PS) composites by in-situ reduction following high-pressure solid phase formation. Reprinted with permission from Ref. [20]. **E** Schematic diagram for the fabrication of chemically modified graphene/polyimide (CMG/PI) nanocomposites with laminate structures by electrospinning–hot pressing method. Reprinted with permission from Ref. [21]

Functional polymer composites (FPCs) have gained enormous attention in recent decades owing to their great potential in delivering a wide range of functionalities, e.g., electrical conductivity [1–3], strain sensing [4, 5], thermal conductivity [6–8], thermoelectric property [9, 10], dielectric property [11, 12], electromagnetic shielding (EMI) [13–15] and microwave absorption [16]. These functionalities are largely determined by physicochemical properties of functional fillers (i.e., size and shape of fillers) and their network morphology in polymer matrices. Therefore, as shown in Fig. 1, numerous research efforts have been devoted to the optimization of these functionalities through morphology control and interfacial modification, where a wide range of preparation methods and exciting performance of FPCs have been reported [17–21]. Despite the fact that these FPCs have many similarities because they all consist of functional inorganic fillers and polymer matrices, reviews on the progress of FPCs are limited in individual FPCs field, while overview on the overall progress of FPCs is stilling lacking, and especially the overall processing strategies for these composites is urgently needed.

As well known, the structure–property relationship is a key guidance in designing polymeric composites. Furthermore, tailoring structure of FPCs by morphology control and interfacial modification finally footholds on the selection and combination of preparation methods in practice. In this regard, appropriate selection and combination of preparation methods is a prerequisite to enable the optimization of functionalities. To date, most of researchers merely focus on the morphology control of filler network and modification of the interface between filler and polymer [22, 23], while the relationships between different processing methods, structure of FPCs (including filler network morphology and interface between filler and polymer) and final functionalities are still not clear. Meanwhile, researchers tend to focus on their own specific field [24]. Nevertheless, finding new inspiration from other mainstream or specific processing methods in different FPCs fields may pave a novel potential way for the preparation of high performance FPCs.

However, there are many processing methods for preparing FPCs, not to mention the large number of combinations between different methods. It is actually not easy to select the most suitable processing methods for wide range of FPCs. Therefore, efficient classification and summary strategy is vital to systematically study processing methods and processing method–structure–property relationship. After considering above issues, the concept of “Toolbox” is proposed based on regarding one or several similar processing methods as a kind of tool for designing various FPCs. Previous work from our group with some specific tools for the morphology control “Toolbox” has been reported [25]. To extend the tools for various FPCs and summarize the “Toolbox” more comprehensively, the processing and fabrication of various FPCs are summarized for wider range of literature. Then, a systematic “Toolbox” is developed, which covers recent progress on processing methods for better designed functionalities as shown in Table 1. To better understand these processing methods, they are firstly categorized into various processing types: melt blending, in situ polymerization and solution blending. Furthermore, specific processing methods are divided into primary processing tools and secondary processing tools according to the sequence they are utilized, which will be listed in detail in Sect. 2. Thereinto, primary processing tools include hybrid filler, volume excluding particle, polymer blends and filler surface modification, while secondary processing tools have more species, such as foaming, thermal annealing, injection molding, magnetic field, coating, film casting, pre-straining and electrospinning. Therefore, such “Toolbox” could be used to not only study the relationship of processing method–structure–property, but also possibly create novel approaches for the preparation of certain FPCs by combining a number of exiting tools, or adapting tools from other type of FPCs. Meanwhile, newly emerging designed processing tools could also be incorporated into such “Toolbox” to update and enrich the system.

**Table 1:**
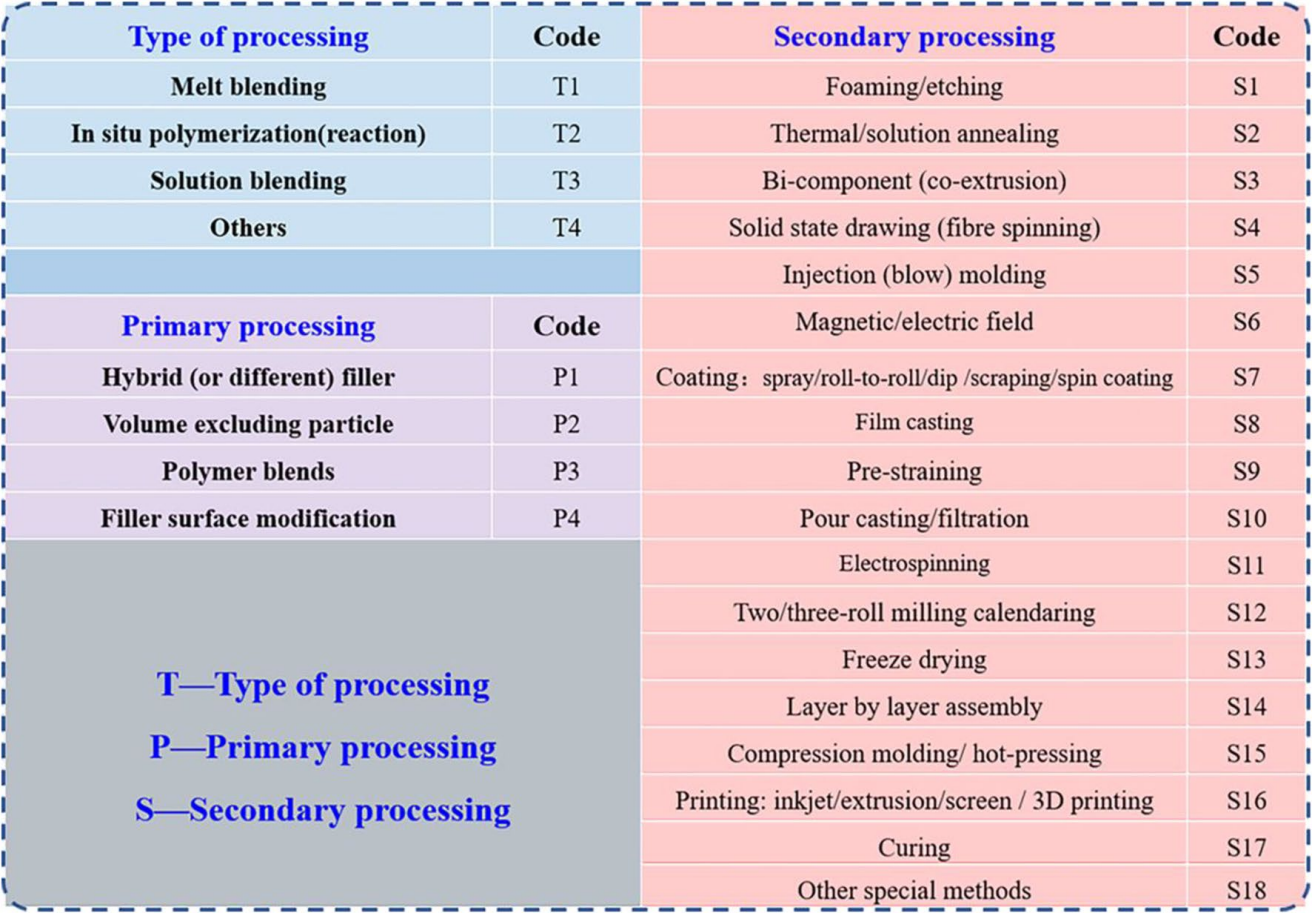
“Toolbox”: the type of processing and specific tools used during processing for morphology control. These tools are summarized from 700 literature collected in various FPCs fields

This review provides a comprehensive overview on the to-date processing methods for preparing FPCs, including electrically conductive polymer composites (ECPCs), strain/pressure sensing polymer composites (SSPCs), thermally conductive polymer composites (TCPCs), dielectric polymer composites (DEPCs), electromagnetic interference (EMI) shielding polymer composites (EMISPCs), microwave absorbing polymer composites (MAPCs) and thermoelectric polymer composites (TEPCs). The basic mechanism and background for above FPCs can be found in Supporting Information and a number of decent review papers [23, 26–34], respectively. In this review, we start with the details and characteristics of different processing methods and then summarize diverse selections and combinations of processing methods. Furthermore, the structural features brought to the filler networks by these processing methods are also discussed. Additionally, the future application trend of such “Toolbox” is prospected. By combining with “artificial intelligence” computer program, such concept could be used to collect and summarize the overall materials database, and thus, possible processing routes for given structure or targeted properties could be predicted. Such method could be used to guide the future preparation of FPCs to achieve outstanding performance more efficiently and effectively.

## Toolbox

Utilizing inorganic filler and polymer could bring composites with exciting functional properties. However, converting the potential properties of fillers and polymers into composites is not easy. Potential filler agglomeration, filler network distribution in the polymer matrix, the interface between filler and polymer, and the morphology and structure of the entire composite would largely influence the final properties, which pose more stringent challenge to the design strategy and processing method. In the past decade, a large number of reports were focused on morphology control to improve the performance of various composites. This process is realized by different processing methods either individually or collectively. On the one hand, different processing methods have unique processing conditions or implementation methods, and their principles, methods, influencing issues are different to result in different outcome. On the other hand, different processing methods may also lead to similar morphology. For example, porous materials could be achieved by foaming, freeze-drying or even 3D printing. Herein, a concept of “Toolbox” for the processing of FPCs is proposed to summarize and analyze the overall processing strategies for a range of FPCs. As shown in Table 1, the “Toolbox” consists of three parts: the type of processing, primary processing and secondary processing. These types of processing are the major type of processing methods summarized for different literature. Then, primary processing methods can be considered as the materials selection and mixing procedure before processing. Finally, secondary processing methods process various materials into desired shape and structure. All these methods have important influence on the final structure and properties of FPCs. The methods and characteristics of these processing strategies are discussed in the following part.

### Type of Processing

#### Melt Blending

Melting blending is based on the fact that thermoplastic polymers become soft upon being heated up, while the properties of polymers are largely remained the same after they are cooled down. This technique requires relatively high processing temperature; typical amorphous polymers and semi-crystalline polymers can be processed above their glass transition temperature and melting temperature, respectively. Such process can be achieved by equipment, such as extrusion, internal mixing, injection molding and blow molding, which are capable of being operated at elevated temperatures and generating high shear forces. Due to simplicity and availability of these processes, melt blending is considered as the most time-saving, cost-effective and scalable method for the production of FPCs for a wide range of polymer matrixes. This approach is free of solvents and contaminants, which are present in solution processing and in situ polymerization. Nevertheless, the main advantages of those two processing types are that the filler dispersion level could be achieved at molecular scale and provides an advantage of low viscosity, which can facilitate mixing and dispersion. Therefore, compared to solution mixing and in situ polymerization, the filler dispersion status of FPCs with melt blending is often less homogeneous. During mixing, a number of parameters such as temperature, rotation speed and mixing time must be fine-tuned to optimize the resulting properties [35]. During melt blending process, filler agglomerates generally undergo dispersion by erosion and rupture mechanisms simultaneously.

#### In Situ Polymerization

In the process of preparing FPCs by in situ polymerization, the functional fillers are uniformly dispersed in polymer monomers firstly, and then an initiator is added in the filler/monomer system to initiate polymerization. The fillers often need to be surface modified with functional groups or polymer, so that there is a strong interface interaction between fillers and polymer matrix in composite prepared by in situ polymerization. The advantage of in situ polymerization is that inorganic fillers have rather homogeneous dispersion and potentially good interfacial strength between filler and polymer matrix, for which polymer molecules are either wrapped around or covalently bonded to these fillers. Moreover, in situ polymerization enables the grafting of polymer chains onto the surface of fillers, allowing the resulting FPCs with high filler loading level and excellent miscibility with polymer. In situ polymerization is usually used to process FPCs containing thermally unstable or insoluble polymers, which cannot be prepared by other strategies such as solution or melting compounding. Depending on monomer molecular structures, the required molecular weight and molecular weight distribution of the polymers, anionic, radical, ring-opening and other polymerization reactions have been used. Moreover, with the continuous research and the development of in situ polymerization technology in FPCs, many methods, such as in situ intercalation polymerization of polymer in clay [36], template-directed in situ polymerization [37], in situ oxidation polymerization in reverse microemulsion [38] and interfacial adsorption-soft template polymerization [39], are developed to obtain unique structure and high-performance composites.

#### Solution Blending

Compounding techniques, like melt blending, can lead to industry-scale preparation, but it is difficult to realize locally homogeneous dispersion due to the filler–filler interaction, especially for fillers with high aspect ratio such as carbon nanotubes (CNTs), while in situ polymerization is required in relative high-viscosity systems. Thus, another technology, solution mixing, needs to be introduced for better filler dispersion. The solution blending method refers to dispersing inorganic filler in polymer solution using a suitable solvent and removing the solvent finally to obtain FPCs. In the solution blending system, uniform filler dispersion in polymer solution and lower viscosity are two typical aspects. Nanofillers usually cannot be directly dispersed well in most of the solvents due to their exceptionally high specific surface area resulted strong interactions [40]. In order to promote the dispersion of fillers, some additives were introduced in solution blending, such polydopamine and ionic liquid. Deng et al. reported a ionic liquid of 1-butyl-3-(4-sulfobutyl) imidazolium trifluoromethanesulfonate was added into single-walled carbon nanotube/poly(3,4 ethylenedioxythiophene):poly-(styrenesulfonate) (SWCNT/PEDOT:PSS) system [41]. On the one hand, the introduction of ionic liquid promotes the dispersion of SWCNT, and on the other hand, ionic liquid has ion-exchange effect which plays a similar role of high-boiling solvent effects to provide primary and secondary doping of PEDOT:PSS, to enhance the electrical conductivity via manipulation of the ordering structures of conducting polymers. Here is another way to adjust composite morphology and structure by blending filler and polymer matrix in solution blending. Liang et al. reported a morphology design strategy by mixing SWCNTs and polypyrrole (PPy) nanowires, and unique sandwiched layer structure free-standing PPy/SWCNT composite films were obtained [42].

Solution blending can be used to fabricate FPCs with excellent dispersion, and it is also a necessary step for most of the secondary process processing methods proposed such as coating, electrospinning [43] and printing technology [44], but the use of solvent retards their industrial scale production to some extent. There are a number of issues that could be adjusted during this processing, such as the type of solvent, type of shear applied during mixing, mixing sequence, choice of polymer as well as filler, rate of solvent evaporation. These issues could have important influence on the final structure and properties of various FPCs.

#### Others

Other types of processing mainly involve physical mixing method of polymers and fillers and the preparation of thermoset polymers, such as epoxy resin. Physical mixing was widely used to fabricate FPCs with selectively distributed functional nanofillers, i.e., segregated structure. In segregated FPCs, fillers are primarily located at the interfaces of polymeric granules instead of homogeneously distributed. The formation of segregated filler networks substantially relies on polymer matrices with exclusionary microstructure, where functional fillers are located in a constrained volume, thus enhancing the effective density of the filler network at limited loadings [45, 46]. For thermoset polymers, such as epoxy resins, unique epoxide functional groups can react with a vast of hardener or curing agents at an elevated temperature to construct multiple cross-linking networks, resulting in good adhesive, mechanical, chemical and temperature resistance properties [47, 48].

### Primary Processing Method

#### Hybrid (or Different) Filler

**Fig. 2:**
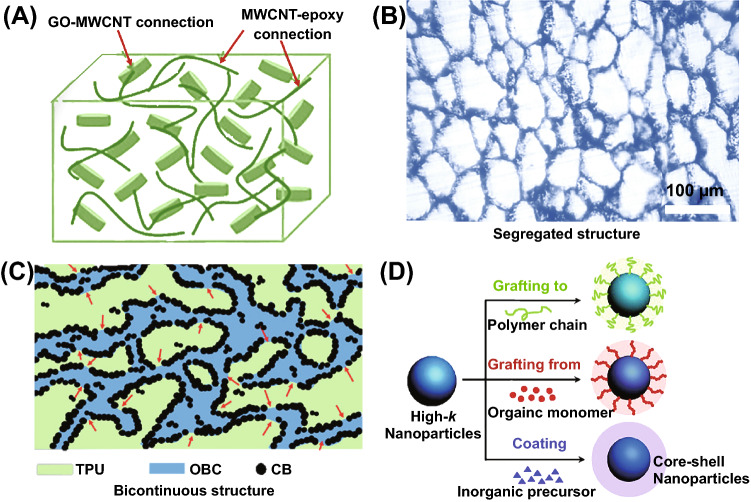
**A** The construction of network structure with hybrid fillers containing graphene oxide/multi-walled carbon nanotube (GO/MWCNT)/epoxy composite. Reprinted with permission from Ref. [50]. **B** Filler particles are localized at the boundaries between the polymer grains to form segregated system. Reprinted with permission from Ref. [53]. **C** Graphical representation of the network morphology evolution for olefin block copolymer/carbon black/thermoplastic polyurethane (OBC/CB/TPU)- ternary blends composites. Reprinted with permission from Ref. [56]. **D** General methods associated with filler surface modification. Reprinted with permission from Ref. [62]

The hybrid filler method refers to adding two or more types of fillers into polymer. On the one hand, the simple superposition of multiple fillers could allow the composites to have the performance of all fillers at the same time, thereby achieving multiple functions. For example, mixing nano-barium titanate and nickel hydroxide can prevent the agglomeration of nano-barium titanate, thereby adjusting the contradictory relationship between dielectric constant and loss in dielectric materials [49]. On the other hand, with the combination of different dimensional topography fillers, a special structure is achieved, thus obtaining a synergistic effect. For instance, single-walled CNTs (MWCNTs) can effectively serve as thermal conduction bridge between graphene oxide (GO) and three-dimensional particles, reducing thermal resistance. As shown in Fig. 2A, MWCNTs which around the GO nanoplatelets can lead to the formation of a heat transport path. Moreover, the MWCNTs on the GO surfaces illustrate synergistic effect on the thermal transport properties due to their 3D network structures [50]. It can be seen that the main characteristic of hybrid (or different) filler is to use fillers with different dimensions, such as 0D and 1D linear, one-dimensional linear and 2D sheets, to build a more complex 3D spatial structure.

#### Volume Excluding Particle

As the name implies, volume excluding effect means that due to the addition of fillers, one of the fillers occupies relative large volume and thus illustrates repelling effect on the other components in the system. This results in the other filler exhibiting some special distribution, thus achieving special functionality. This repulsive effect usually requires a large size difference or shape difference for the filler. At present, this method is mainly used to prepare segregated structure or selectively distributed network, so as to achieve low filler content and high performance. For example, using this method to construct a segregated structure can effectively decrease the filler content needed for achieving conductive networks [51, 52]. For instance, after expanded graphite and ultra-high molecular weight polyethylene (LLDPE) are mixed together by grinding and crushing, compression molding process is used to construct segregated structure (Fig. 2B) [53]. The percolation threshold of such structure is reduced, and other comprehensive properties of the composites can also be optimized [54].

#### Polymer Blends

Polymer blends method refers to two or more polymers that are mixed together to achieve functionality and performance improvement through constructing certain phase morphology. Common polymer blends structures include island-in-the-sea structures, bi-continuous structures, interpenetrating network structures and multilayer structures [55]. It is well known that the phase morphology of a mixture is mainly influenced by two factors, namely thermodynamic factors and kinetic factors. Thermodynamic factors include compatibility and interfacial tension between two phases. It is often observed that better diffusion and uniform mixing among different phases is achieved with better compatibility and greater binding force. Methods to promote compatibility mainly include chemical modification, addition of compatibilizers and formation of interpenetrating network or hydrogen bonding. By controlling process parameters (flow parameters, solvents, temperature, etc.), the phase transition process can be achieved. This special phase morphology brings large performance difference in polymer materials. For example, after adding CNTs to thermoplastic polyurethane (TPU) and olefin block copolymer (OBC) blends with bi-continuous structure (Fig. 2C), composites with enhanced strain sensitivity can be effectively obtained [56]. In addition, it is more noteworthy that structurally special blends, such as multilayer extrusion, multilayer injection molding and gradiently distributed filler, could also be obtained using some special processing methods and instruments. The disadvantage is that the controllable range of properties are often between two blended polymers, and the controllability decreases when multiple materials are used. It should also be mentioned that the use of more than two types of polymers can sometimes generate interesting phase morphology, such as the core–shell structured blends reported by Yang et al. [57, 58]; it could be interesting for a range of applications.

#### Filler Surface Modification

Filler surface modification refers to changing the original inert interface on filler surface through physical or chemical approaches, thereby altering the interfacial interaction between filler and polymer matrix. Generally, the filler surface modification can reduce defects caused by original interface, enhancing the interaction between filler and matrix, promoting the dispersion of fillers in polymer matrix, thereby preventing the performance of composites from degrading. Hence, the methods of filler surface modification can be divided into chemical modification and physical modification. Chemical surface modification is the most commonly method, and it is generally used to modify the filler surface by grafting polymer chain, block polymerization, encapsulation polymerization to filler (“grafting to”) [59]; group decoration or coating organic polymer on filler surface (organic surface modification) [60]; inorganic particles mainly use van der Waals force, hydrogen bonding, chemical bonding, ionic bonding and other methods to strengthen the two-phase interaction (inorganic functionalization), for which popular methods used include: deposition method, outer layer modification, mechanochemical modification, surface chemical modification and high-energy surface modification [61]. Figure 2D shows three typical methods associated with filler surface modification [62]. The purpose of organic phase interface modification in chemical method is to increase the surface polarity and roughness, adjust the surface area and crystallization performance, eliminate weak interface layer, and introduce new functional groups. Related reviews with more details can be found elsewhere [63, 64]. Physical surface modification is usually the use of physical means such as physical adsorption, physical surface functionalization (such as ball milling and plasma treatment) to modify filler surface.

### Secondary Processing

The secondary processing method directly affects the final structure and morphology of the composite. Both structural design and direct shaping to the composite products can be achieved during this process. In order to better understand, secondary processing technologies are categorized according to the final obtained structure and morphology as homogeneous structure, orientation structure, porous structure, layer structure, and others. The homogeneous structure contains S2 thermal/solution annealing, S7 coating, S8 film casting, S15 compression molding and S16 3D printing; the orientation structure contains S4 solid-state drawing, S5 injection molding, S6 magnetic/electric filed, S9 pre-straining, S12 two/three-roll milling calendaring and S16 3D printing; The porous structure contains S1 foaming/etching, S10 filtration, S11 electrospinning S13 freeze drying and S16 3D printing; The layer structure contains S3 Bi-component(co-extrusion), S14 layer by layer and S16 3D printing. S17 curing and S18 few special processing tools are not classified here because of the particularity of their use. Moreover, distinguishment of these secondary processing methods is discussed in the each S tool. In this section, we summarize various recent progress on processing methods according to their similarity and give an overview of their principles, classification, usage methods and scenarios.

#### (S1) Foaming/Etching

**Fig. 3:**
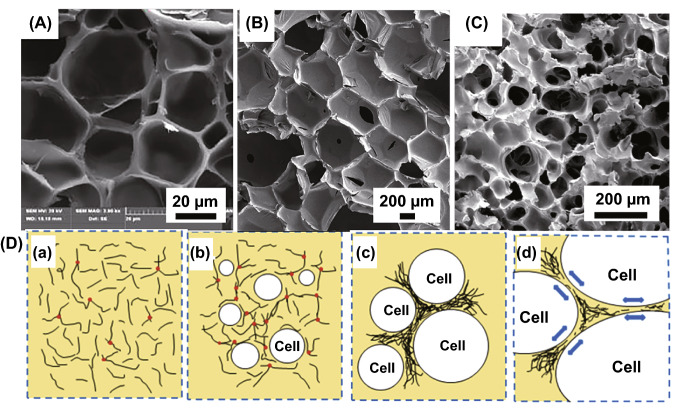
Cellular structure of polymer foams: **A** closed-cell foam. Reprinted with permission from Ref. [69]. **B** Partially open-cell foam and **C** open-cell foam. Reprinted with permission from Refs. [18, 70]. **D** Schematic diagram of the microstructural changes foaming process with the growth of cells. The black lines represent the filler, and the arrows show the growth directions of the bubbles during foaming. Reprinted with permission from Ref. [71]

Polymer foam has the advantages of light weight, good heat insulation, sound absorption and shock absorption, and they have been widely used in military and civilian industries. It is commonly classified into three types [65]: closed-cell foam, partially open-cell foam and open-cell foam. Figure 3A–C corresponds to the cellular structure of polymer foams. Generally, the basic step of foam forming is to have a bubble core, which is called nucleation, then the bubble core grow or expand in polymer melt, finally diffuse out. The driving force present in forming process is gas over-saturation caused by increasing temperature or decreasing pressure. The schematic diagram of the microstructural changes foaming process with the growth of cells is shown in Fig. 3D. More details about nucleation can be found elsewhere [66]. Polymer-based foams can be prepared using physical or chemical foaming. Physical blowing agents act on polymer matrix as inert occupation. For instance, some low-boiling-point agents act as physical blowing agent by dissolving and evaporating from polymer, such as pentane and freon [67]. Considering air pollution, environment-friendly physical foaming agents including carbon dioxide, nitrogen, air and water have been used for foaming. This method starts from the saturation of polymer filled with uniform concentration gas at elevated pressures, followed by heating and reducing the pressure to obtain cell nucleation. Kuang et al. reported a pressure-induced physical foaming method by supercritical carbon dioxide (Sc-CO_2_) to prepare “brick and mud” structure L-polylactic acid (PLLA)-MWCNTs composites [18]. The saturated Sc-CO_2_ filled in PLLA-MWCNTs block in a high pressure at certain temperature for a restricted state, then cool down and release them to induce the cell nucleation and bubble growth to achieve uniform foaming. Chemical blowing agents take part in reaction in foaming process and giving off gas to form cell, so foam-making process is irreversible. Isocyanate and water are often used in PU foaming, azohydrazine and other nitrogen-based materials in thermoplastic and elastomeric foaming, and sodium bicarbonate in thermoplastic foaming. For example, Covavisaruch et al. employed azodicarbonamide (AC) in foaming polyvinyl chloride (PVC)/rice hull composites and investigated the effect of concentration and particle sizes of AC on density and cell size. With increasing foaming agent content, the cell size became smaller [68]. For industrialization, extrusion foaming and injection molding foaming technology are used for large-scale production of polymer-based composite foam [65].

During the processing of FPCs, etching is generally used to construct voids or rough surface. It is divided into dry etching and wet etching. Dry etching refers to the use of laser or plasma, generally in order to form rough surfaces and then functional modification. For example, Xu et al. used low-pressure oxygen-plasma treatment to increase the roughness of PU backbone and activate the surface of original PU foam backbones by introducing abundant oxygen-containing groups. In this work, oxygen plasma is used as an etchant to physical bombard the surface of object through insert, generate or remove functional groups [72]. Meanwhile, wet etching refers to the use of chemical solution to etch the interior of materials, accompanied by chemical reactions and the formation of pore structure. For example, Pang et al. grew multilayer graphene on nickel foam and formed polydimethylsiloxane (PDMS) coating by immersion graphene/Ni foam into PDMS prepolymer. Then, they employed HCl to etch the nickel skeleton to obtain porous structure composites, and an interconnected graphene network was obtained. This porous composite demonstrates good flexibility and can undertake bending, torsion and stretch [73].

#### (S2) Thermal/Solution Annealing

Thermal/solution annealing is an effective method for promoting the increase of crystallinity and morphological control in FPCs. Through such process, the polymer matrix was heated above melting temperature or glass transition temperature, accompanying the relaxation of their molecular chains. Through the relaxation and mobility of polymer molecules, the functional fillers are redistributed and the filler network structure is formed. In addition, residual stress could be eliminated. Generally speaking, the crystallinity is often increased after thermal annealing, which would often lead to changes in the mechanical properties of FPCs, such as the elastic modulus and yield strength.

In inorganic fillers/polymer systems, thermal/solution annealing could promote the formation of conductive networks in FPCs containing various types of conductive fillers. Zhang et al. showed that conductive microfibrils are separated by oriented polyethene (PE) crystals [74]. The segmental Brownian movement of PE chains facilitates the disordering of microfibrils during annealing process, in which overlapped microfibrils created new conductive paths and induced dynamic percolation transition. Furthermore, thermal annealing also has important influence on the orientation of conductive fillers. In our previous study [1], morphological control of conductive network was realized by solid-state drawing bi-component structure tape followed by annealing between the melting temperatures of different components. During annealing, highly oriented MWCNTs was relaxed with much better local contacts between still largely oriented MWCNTs bundles. In addition, another thermal/solution annealing method is sintering, where sintering temperature is usually quite high to allow the diffusion between polymer particles [75]. The influencing issues during sintering could be: sintering temperature, sintering time, pressure, particle composition, etc. Generally, thermal/solution annealing is often combined with other technologies to achieve more effective functional design, such as electric/magnetic field, solid-state drawing, injection molding.

#### (S3) Bi-component (Co-extrusion)

Bi-component (co-extrusion) is an important processing method to prepare multicomponent FPCs. In this process, polymers melt and fillers with different properties are added to the extruder and extruded with a certain shape of die under extrusion. Bi-component (co-extrusion) technology is applicable to almost all thermoplastic and some thermosetting plastics such as phenolic resin. With the advantages of simple process, energy saving, high production efficiency, greatly reduced production costs and no solvent is used in the process. Through co-extrusion, the interface between polymers produces adhesion, the adhesive force between different polymer resin composite layers mainly depends on the mutual solubility and affinity of resin matrix, and the melting temperature difference between different resins should not be very large. Additives for adhesion could be incorporated between polymer layers during co-extrusion if necessary. Meanwhile, component viscosity match, rheological behaviors, temperature control, extrusion speed and other instrument parameter design are also the key factors affecting the preparation of high performance FPCs during co-extrusion. It should be pointed out that there is a “viscosity surrounding” from low-viscosity melt to high-viscosity melt. If the die flow channel is long enough, the phenomenon of “viscosity surrounding” would lead to uneven layer thickness distribution in the final composites. Moreover, the melt with higher viscosity would be completely coated by the melt with lower viscosity. In addition, because of the different viscoelastic properties of various polymer melts, the interlayer interface of the melt is easily formed by co-extrusion, producing "wavy" or "zigzag" instability.

To have more control on the multilayered structure in various polymer films, Guo et al. fabricated alternating multilayered polypropylene (PP) and carbon black (CB)-filled polypropylene (PPCB) composites through layer-multiplying co-extrusion system and investigated its electrical behaviors [76]. By controlling the co-extruding speed and the die setup, the number of layers and thickness of each layer could be controlled. These alternating multilayer composites exhibit anisotropic electrical behaviors because insulating PP layer would hinder the establishment of conductive pathways. Moreover, the alternating insulating PP layers and conductive layers vertical to interfaces could accumulate electrical charges at the interface, leading to significantly enhanced dielectric permittivity. More details on co-extrusion can be found in review paper by Zhang et al. and Li et al. [77, 78].

#### (S4) Solid-State Drawing/Fiber Spinning

Solid-state drawing is often carried out on materials between their glass transition and melting temperatures [79]. A suitable drawing temperature has important influence on molecular rearrangement and crystallization. As the results of drawing, modulus, strength and toughness can be improved by the strain-induced crystallization and orientation [80]. Apart from drawing temperature, draw ratio is another key factor on the mechanical properties of polymer composites.

Fiber spinning is a method to form fiber, including melt spinning, dry spinning, wet spinning, and dry-jet-wet spinning, etc. [81]. Melt spinning is one of the most widely used processes in fiber manufacturing. During melt spinning, polymers are drawn over its melt temperature thanks to the high molecular mobility of polymer melt. There is a large temperature gradient between fiber and surrounding environment. The strong shear field forces the polymer to stretch and form into filament rapidly under ambient temperature difference, and inorganic filler is often highly aligned along the spinning direction within polymer matrix. Spinning temperature, spinning rate, subsequent hot-drawing process are important factors affecting fiber performance, surface quality and crystal structure of the fibers [82]. Other factors affecting the final functional properties are the dispersion of inorganic fillers in the fiber, filler content and filler network structure, etc.

Dry spinning is a method similar to solid-state drawing and melt spinning. When the precursor polymer solution is extruded from the needle, the external evaporating hot air attacks the fiber and the solvent evaporates leaving a filamentous fiber. In the process of wet spinning, polymer composites solution is extruded and passes through a liquid precipitation bath to form fibers. And the extruded solution passes through an air route before entering a precipitation bath in dry-jet-wet spinning. Moreover, the factors affecting fiber spinning in processing are: viscosity, temperature, extrusion rate, cooling conditions (stretch ratio, precipitation conditions), etc. Overall, oriented polymer and filler network structures with different degree of alignment are often obtained during above drawing and spinning process.

#### (S5) Injection (Blow) Molding

During injection molding, polymer melt is stirred by a screw, injected into the mold cavity with high pressure, and then cooled and solidified to obtain product at certain temperature. In its routes, there are three main steps in the molding: filling, packing/holding and cooling [83]. Various types of polymers with desired flow properties and a low viscosity at high temperatures are suitable to produce injection molding parts, such as polystyrene (PS), polymethyl methacrylate (PMMA), PP, PE and acrylonitrile butadiene styrene (ABS). It is noteworthy that final impact on processing decision would not only depend on type of polymers but also temperature control, pressure control, injection speed and many other conditions in the process [84].

Composites exhibit a variety of microscopic morphologies under shear and temperature fields employed by injection molding, and the orientation of fillers and polymers along the injection molding direction have received widespread attention. The orientation structure of the sample is closely related to the shear field during injection molding. Due to the difference in shear force, the structure in the area close to the cavity wall (skin layer) and the center area (core layer) is significantly different [85]. For ECPCs, the composites obtained by injection molding generally have a larger percolation threshold compared to compression-molded composites [86]. Meanwhile, more confinement on polymer composites could be achieved with decreased mold thickness and increased injection rate. For instance, Yu et al. observed such confinement could deform polymer blends into multilayered structure, resulting in alternating multilayer CNTs conductive networks in polyolefin blends through high-speed thin-wall injection molding [79]. Nowadays, the trend of processing technology is developing toward microinjection, high fill compound injection, water-assisted injection molding, mixed use of various special injection molding processes, foam injection molding, mold technology, simulation technology and so on [87].

Blow molding is a method that the polymer melt is placed in the split mold, and compressed air is introduced to make the mold embryo cling to the inner wall of the mold, which is cooled and demolded. Similar to injection molding, blow molding is also forced by shear. Compared with injection molding, blow molding products are inflated under low pressure, so the residual stress of products is smaller, and the performance in tensile, impact, bending and environmental strain resistance is often better.

#### (S6) Electric/Magnetic Field

Electric and magnetic fields are effective assistant method to assemble functional fillers in polymer system. Under external electric or magnetic field, filler migrates forced by electric or magnetic field, which reconfigure the filler network. For instance, alternative current (AC) electric field was used to induce nanofibrillated cellulose (NFC) alignment in TPU system in order to enhance the dielectric and mechanical properties [88]. Induced by electric field, NFC is easily orientated because of electric field-induced polarization, which induced a torque to force NFC along the electric field direction. Consequently, the prepared anisotropic TPU/NFC composites exhibit increased tensile strength and elongation at break in the parallel direction than that in the vertical direction. Magnetic field was also used to assemble filler with the participation of magnetic components, such as magnetic fibers or particles. Chen et al. [89] mixed solution of Co nanowires and polyvinylidene fluoride (PVDF) by drop casting onto aluminum sheet with magnetic field to enforce orientation, then the Co nanowires would assemble into highly ordered domains. Along the unidirectional desired direction, the electrical transport in oriented Co nanowires can be greatly promoted, and also the filler content required to form a percolated network can be possibly reduced. The results show that oriented nanocomposites exhibit significantly increased electrical conductivity than identical nanocomposites that are randomly oriented. Another strategy is that when magnetic particles adhere to the surface of filler, the magnetic particles drive the filler to orient under magnetic field. For instance, magnetic Fe_3_O_4_ nanoparticles on the surface of a certain type of filler could guide filler re-orientation under external magnetic field [90]. Overall, such method based on external fields often result in filler structure with some degree of orientation without significantly orienting polymer matrix. Meanwhile, better filler local contacts are also often observed.

#### (S7) Coating: Spray/Roll-to-roll/Dip Coating/Scraping/Spin Coating

Coating is one of the most widely used processing methods for fabricating functional films or layer of functional coatings on various substances, such as fiber, foam, electrospun mat, film or any solid surface. Wide range of filler contents could be achieved in functional polymer composites in coating processing. According to the filler distribution in these films or any other substances, two different systems can be identified. One of them is the most adopted method: the functional filler is evenly distributed in the polymer system and then coated onto a given substance. This method has been adopted for functionalities including electrically conductivity, thermoelectric, dielectric, thermal conductivity, EMI shielding and sensors. For instance, Hong et al. [91] fabricated high-performance thermoelectric nanocomposite films as organic thermoelectric generators through spray coating a mixture of CNTs and poly(3-hexylthiophene) (P3HT). Surface modifier is often introduced into the mixed system. For instance, Shen and coworkers [92] utilized dopamine (PDA) to modify h-BN microplatelets and stirred with polyvinyl alcohol (PVA) aqueous solution, where the PDA coating increases the dispersibility of the filler and enhances its interaction with PVA matrix. Followed by scraping, the hexagonal boron nitride (h-BN)/PVA composite film was achieved.

The other is the multiple coating strategy, where separate steps were used to prepare filler and polymer layers, respectively. For example, Liang et al. presented a PDMS/Ag nanowires composite flexible transparent conductive film by spin-coating PDMS onto Ag nanowire network [93]. An Ag nanowire (AgNW) network was fabricated firstly via spin-coating of AgNW solution onto a polyethylene terephthalate (PET) substrate, and then the obtained AgNW network was placed on HCl vapor under visible light for nanowelding. Finally, PDMS layer was spin-coated onto AgNW network to obtain PDMS/AgNW film. This strategy not only constructs controllable AgNW welding network, but also evidently enhances the conductivity. Meanwhile, dip coating is the same interface contact mode between filler and polymer. Kim et al. firstly dipped basalt fiber in MWCNT dispersion to prepare conductive composite filler, then the MWCNT/basalt fiber was immersed in epoxy to obtain the final MWCNT-coated basalt fiber/epoxy composites [94]. Through this method, the electrical conductivity of such MWCNT-coated basalt fibers epoxy composites increases significantly from 3.25 × 10^−9^ to 1.44 × 10^−1^ S cm^−1^ with increasing dip-dry coating cycle from 0 to 10.

Overall, various coating methods have been widely used in industry to produce a variety of functional composites. These coating methods could be divided into spray coating, dip coating, comma roller coating and slot die coating. They are selected for applications according to the specific requirement and solution properties including solid content, viscosity, bubble generating ability during coating and sedimentation of solid [95].

#### (S8) Film Casting

Film casting is a method to prepare FPCs film by casting the mixture solution of functional filler and polymer into substrate followed by the evaporation of solvent. In this process, the mixture solution is spread out by surface tension to form a thin film. Besides, there is usually no obvious orientation structure that emerges in casting process. For instance, Xu et al. [96] prepared conductive PMMA/CFs (carbon fibers) composite films by casting different concentrations of CFs mixture solution on a rectangular glass plate to obtain composite films. The CFs are overlapped and disorderly distributed with each other, and finally form conductive network. Compared with the above coating method, film casting tends to produce films with larger thickness, and unlike the above coating methods, it is often unsuitable for substance which is not flat films.

#### (S9) Pre-straining

**Fig. 4:**
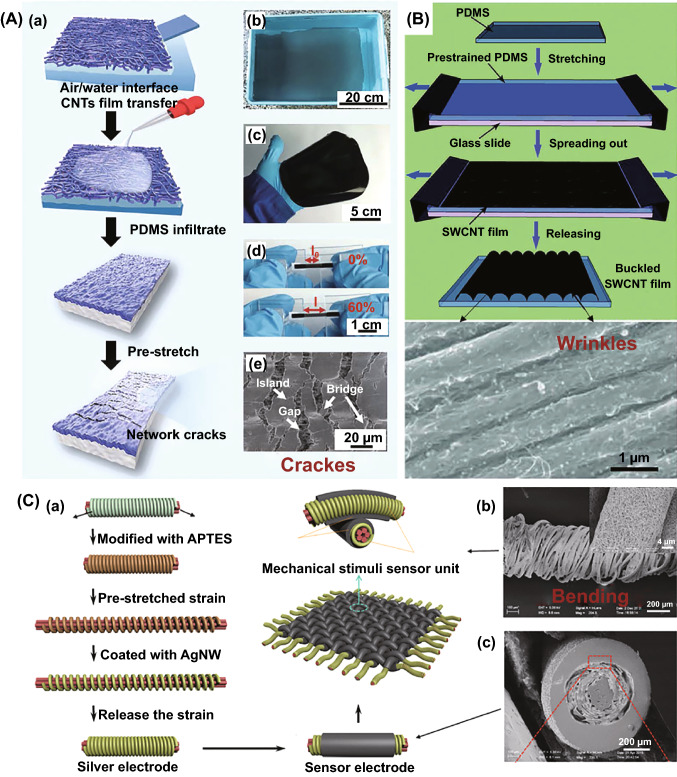
**A** Illustration of the fabrication process of CNTs films/polydimethylsiloxane (PDMS) strain sensors and network cracks after pre-stretching, consisting of gaps, islands, and bridges connecting separated islands. Reprinted with permission from Ref. [97]. **B** Schematic of preparing buckled SWCNT film on PDMS and buckled structure after pre-stretching. Reprinted with permission from Ref. [98]. **C** Schematic illustration of the fabrication processes of the wound around polyurethane fibers core surfaces coating by AgNW with bending structure of the sensor electrode. Reprinted with permission from Ref. [99]

Pre-straining is a method of pre-stretching a given object and then releasing the stress to produce special structures, such as cracks, wrinkles or bending. Through such process, functional filler network can often be oriented during stretching and relaxed during releasing. Such process has important influence on the reconstruction of conductive networks and is often used to prepare flexible and stretchable devices, such as flexible conductors and strain sensors. Wang et al. [97] fabricated a pre-straining followed buckling approach to prepare a high stretchable and sensitive CNTs film/PDMS composite with hierarchical crack structure. The PDMS solution was poured onto the multilayer CNTs film to form CNTs film/PDMS composites and cut into small strips after curing. After the strips were pre-stretched to 120% strain, network cracks were produced in multilayer CNTs film, including gaps, islands and bridges connecting separated islands (Fig. 4A). Niu and coworkers [98] stretched the PDMS substrate to 30% pre-straining and then spread a surface hydrophilic group of SWCNTs film out onto the pre-strained PDMS substrate directly. After releasing the stress, some wrinkles structure would be formed (Fig. 4B). Ge et al. [99] provided a stretchable electronic fabric artificial skin approach with a helical AgNWs network by pre-straining. A 100% pre-straining elastic thread (a nylon fiber helically wound around inner PU fibers core surfaces) was coated with AgNWs solution through dip coating and then a helical AgNWs network (Fig. 4). Thanks to this unique helical structure, the AgNWs networks can still keep the conductive paths and high conductivity even if sustain large tensile strain. Besides, pre-stretching could also be used for conductive polymer composites [100, 101] and thermal conductive polymer composites [102].

#### (S10) Pour Casting/Filtration

**Fig. 5:**
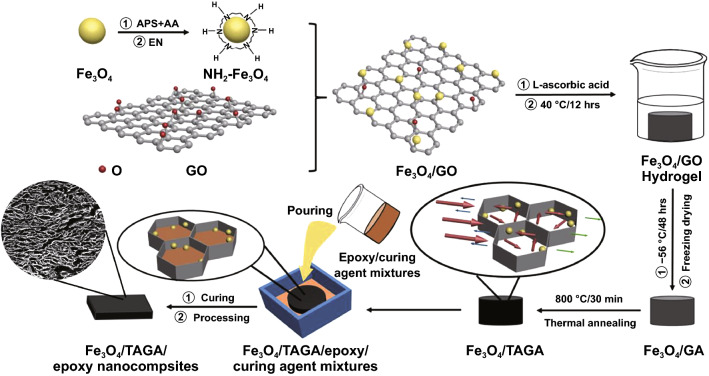
Schematic diagram of the fabrication for Fe_3_O_4_/ thermally annealed graphene aerogel (TAGA)/epoxy nanocomposites by pour casting. Reprinted with permission from Ref. [104]

Pour casting/filtration is a typical combination method for fabricating FPCs. It is generally applicable to fillers with structural framework or fixed shape, such as 3D network structure or fillers fixed in mold. The polymer serves as protective layer and fills the gap among the fillers. For instance, Yu et al. [103] prepared high-performance epoxy nanocomposites reinforced with 3D CNT sponge for EMI shielding by filtrating epoxy into as-prepared porous CNT sponge with a vacuum-assisted method. The excellent electrical, EMI shielding and mechanical properties of epoxy/CNT sponge nanocomposites can be attributed to the more continuous and strong conductive network for electron transport, EMI shielding and load transfer. Generally, pouring casting/filtration method does not damage the structure of functional filler, Gu [104] reported a Fe_3_O_4_/thermally annealed graphene aerogel (TAGA) EMI shielding composite by pour casting epoxy in Fe_3_O_4_/TAGA aerogel. It can be observed that the composite remains the same filler network structure before pour casting (Fig. 5).

#### (S11) Electrospinning

**Fig. 6:**
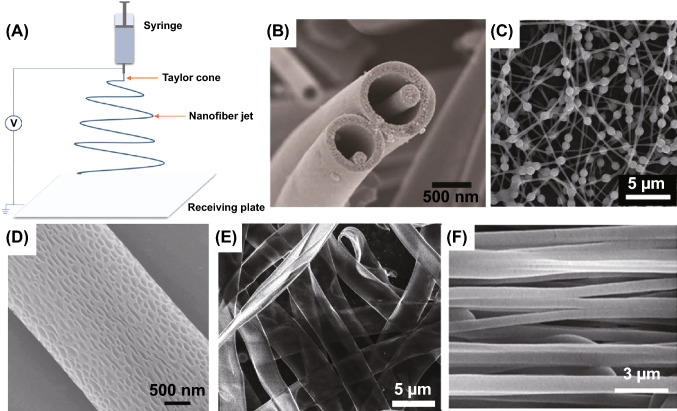
Schematic showing electrospinning setup (**A**) and electrospinning with some special morphology: **B** core–shell fibers. Reprinted with permission from Ref. [106]. **C** Beaded fibers. Reprinted with permission from Ref. [107]. **D** Porous fibers. Reprinted with permission from Ref. [108]; **E** ribbon fibers. Reprinted with permission from Ref. [109]. **F** Oriented fibers. Reprinted with permission from Ref. [110]

Since 1990s, electrospinning has attracted a great deal of interest due to its advantage of simple operation, strong designability and yielding continuous fibers with diameters down to the nanometer scale, having a rapidly development in recent years [105]. Unlike conventional fiber spinning techniques, electrospinning is driven by electrical force, and the polymer solution overcomes surface tension to form jet under high voltage electric field, solidifies into fibers in the air and finally falls onto the receiving plate. Figure 6A is a schematic showing electrospinning setup. This process is complex, and the main influencing factors include the uniform dispersion of filler, solution viscosity, applied voltage, flow rate, collecting distance, and other ambient parameters (temperature, humidity, etc.). And the fiber collected equipment is diverse, leading to various fibrous assemblies [82].

Generally, the functional filler or its precursor is uniformly dispersed in the polymer solution; thus, the filler is embedded in matrix during electrospinning process, which can make the filler realize nano-scale dispersion in matrix. In addition, electrospinning is able to prepare fibers with various morphologies: core–shell fibers (Fig. 6B) [106], beaded fibers (Fig. 6C) [107], porous fibers (Fig. 6D) [108], ribbon fibers (Fig. 6E) [109], and it is also possible to prepare oriented fibers by controlling the speed of the receiving roller (Fig. 6F) [110]. Design for functional properties is often used in electrospinning process, and Guo et al. incorporated NH_2_-POSS-grafted boron nitride and fluorine-containing polyimide into electrospinning fibers for excellent thermal conductivity. This method improves the interfacial compatibility and reduces interfacial thermal resistance between thermally conductive fillers and polymer matrix. And electrospinning is in favor of better contact between adjacent fillers with polymer fibers, leading to improvement in thermal conductivity [111].

#### (S12) Two/Three-roll Milling Calendaring

Two/three-roll milling calendaring is a processing method, in which by means of the strong shear force between parallel rollers, at corresponding processing temperature, viscous materials are squeezed and extended for many times. Two/three milling calendaring can mix functional fillers and polymer matrix effectively. When the mixture pass through the gap between rotating cylinders, materials could be sheared through adjusting cylinders rotating at different velocities who impart high shear stresses. Moreover, because of the large shear stress, functional fillers and macromolecules would be oriented, which makes the film anisotropic in physical and mechanical properties. With the advantages of being solvent-free, uniform shear field, being scalable, and easy handling of high filler loadings, two/three milling calendaring has been used in various applications. For instance, Nam et al. incorporated CNT into epoxy to obtain effective electromagnetic wave absorbing composites films through three-roll milling followed laminating processes [112]. Ravindren et al. blended poly(ethylene-co-methyl acrylate)/ethylene octene copolymer (EMA/EOC) and MWCNTs by solution mixing. After drying, the composites were homogenized by passing through two roll mill subsequently [113].

#### (S13) Freeze Drying

Freeze drying is a method of drying materials using the physical phenomenon that water sublimates gas from ice [114]. After gas sublimation, hole channels are left to form porous structure. Freeze drying is essential for the creation of a dried hydrophilic substance with high specific surface area and pore volume. It is a simple and efficient approach to fabricate materials with low density and high specific surfaces due to the 3D highly porous structures. And as a template free preparation method, it is desirable for scalable manufacturing of aerogels with controllable density and shape.

**Fig. 7:**
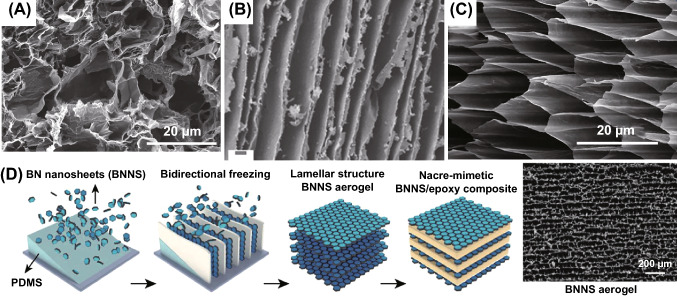
**A** SEM of disordered freeze-dried of cellulose/graphene nanoplatelets. Reprinted with permission from Ref. [115]. **B** SEM of freeze-dried anisotropic porous MWCNT/WPU composites. Reprinted with permission from Ref. [116]. **C** SEM of unidirectional freeze-drying cellulose nanofiber/CNTs/PDMS composites. Reprinted with permission from Ref. [117]. **D** Schematic of the fabrication route using a bidirectional freezing technique to obtain BN/epoxy composites. Reprinted with permission from Ref. [118]

There are various strategies to achieve freeze drying. Ordinary freeze drying usually produces disorder structure as shown in Fig. 7A [115]. Zeng et al. reported a facile freeze-drying method to fabricate an anisotropic porous MWCNT/water-borne polyurethane (WPU) composites for EMI shielding (Fig. 7B) [116]. The obtained MWCNT/WPU composites were anisotropic porous architectures interconnect by cell walls which MWCNTs interspersed into WPU matrix, forming overlapping conductive network. Moreover, freeze drying technology is also a method to rearrange fillers. Wang et al. operated a unidirectional freeze drying method by immersing a precursor tube in liquid nitrogen at a constant speed with glass beads filled in tube bottom. In this system, a honeycomb-like structure with isotropic in directions perpendicular to the channels is formed (Fig. 7C), contributing excellent electrical conductivity and Young’s modulus along the direction of penetrating microchannels. It is approximately twice of those in the orthogonal direction in cellulose nanofiber/CNTs/PDMS composites strain sensors [117]. Bai and coworkers constructed 3D nacre-mimetic BN/epoxy composites conductive network by bidirectional freeze drying. They utilized a low thermally conductive polydimethylsiloxane wedge to generate temperature gradients in both the horizontal and vertical directions, guiding by which ice crystals nucleate and grow into a long-range lamellar pattern (Fig. 7D). At the same time, BN nanosheets were assembled to highly organized 3D conductive network, providing prolonged phonon pathways. Such materials illustrate a high thermal conductivity (6.07 W m^−1^ K^−1^) at 15 vol% BNNS loading and illustrate high anisotropic thermal behavior (*λ*||/*λ*⟘ as high as 12), excellent electrical resistivity (2 × 10^12^ Ω cm), and thermal stability (glass transition temperature 120 °C) [118].

#### (S14) Layer by Layer Assembly

In the past few decades, layer by layer (LbL) assembly has received prosperous development as its simple preparation, controllable thickness and wide range of applications [78]. For the preparation of FPCs, such method is of great significance to the layer structure design and functionalities. Multilayered hierarchical structure, layer interfaces, and morphologies of multilayered polymer composites can be controlled through LbL method. The original LbL assembly technology was driven by electrostatic force as reported by Iler, where polyelectrolyte multilayers were prepared by alternate deposition in polyelectrolyte solution with opposite charges [119]. Since then, LbL assembly method has aroused great research enthusiasm. With years of continuous research, a series of LbL assembly technologies have been developed [78]: driving force by electrostatic force, hydrogen bonding, charge transfer interaction force, covalent effect, host–guest interactions, compression pressure, shearing force and temperature-field driven methods have been proposed to prepare FPCs. In addition, the LbL assembly method is also expanded by strategies of immersive assembly, spin assembly, spray assembly, electromagnetic assembly, fluidic assembly, and so on. For example, Wang et al. [120] fabricated 3D BN nanosheet-wrapped melamine foams (MF@BNNS) by repeated LbL assembly using melamine skeleton as substrate to realize high-efficiency thermal conductivity. As polyethylenimine is positively charged, the MF foam was immersed in polyethylenimine solution for the following electrostatic interactions with negatively charged BNNSs. Then, via the alternate deposition of BNNSs and polyethylenimine multiple times by LbL assembly technique, epoxy resin was vacuum-assisting infiltrated on MF@BNNS foam to prepare epoxy-based thermal conductive composites. Heo et al. [121] used LbL assembly method to prepare three component microwave absorbing composite of poly(acrylic acid) (PAA)/oleic acid-ferrite blend layer and a poly(allylamine hydrochloride) (PAH) layer based on electrostatic interaction. Combined with electrochemical polymerization for a high power factor, Culebras et al. [122] deposited PEDOT on as-prepared MWCNT film in a three-electrode electrochemical cell to achieve LbL assembly with a homogeneous microscopic structure. Besides, LbL-laminated assembly based on pressure, multilayered co-extrusion technology, crystallization and annealing was also applied in polymer melts processing [78].

#### (S15) Compression Molding/Hot pressing

During compression molding/hot pressing, FPCs are compressed at certain temperature under given pressure. This method is simple and highly efficient, which is intensively used in FPCs processing. In general, molding pressing/hot pressing is used as a step in combination with other processing methods [123]. Through hot pressing, the filler and polymer can form a closer interface, and finally the isolation network structure can be obtained [124]. For instance, Yang and coworker have recently reported three different methods (ball milling, freeze-drying and electrospinning) followed by hot pressing were applied to explore the thermal conductive performance based on BNNS/PVA composites films [125]. In addition to forming a filler/polymer tight interface with uniform dispersion of filler, Chen’s group reported composites by solution mixing and subsequent melt pressing to afford high performance with high degree of filler orientation [126–128]. Under hot pressing, the SWCNTs with 1D nanostructure were dramatically aligned by polycarbonate (PC) melt flowing in the radial direction. This study broadens the road to obtain judicious alignment fillers in polymer composites via hot pressing [129]. Overall, processing conditions such as pressure, time and temperature are important influential issues on the structure and properties of the final FPCs, and isotropic composites are often fabricated through such process.

#### (S16) Printing: Inkjet/Extrusion/Screen/3D Printing

3D printing is a popular technology to obtain complex 3D structure devices without the typical waste in recent years [130]. Various printing techniques have been employed to fabricate polymer composites, such as fused deposition modeling (FDM), selective laser sintering (SLS), powder bed and inkjet head 3D printing (3DP), stereolithography (SLA), 3D plotting/direct-write and others new techniques are still in development [131]. Among them, direct-write and FDM technology are the most commonly ways for fabricating FPCs.

**Fig. 8:**
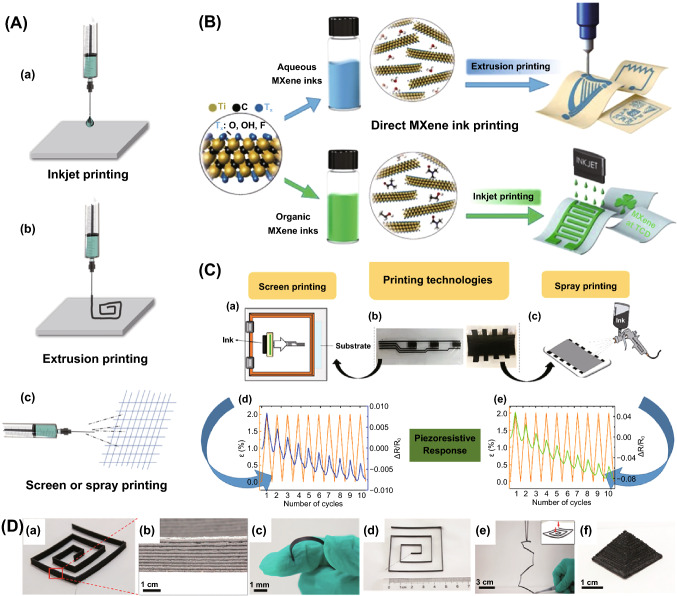
**A** Schematic of different printings: **a** inkjet printing, **b** extrusion printing, **c** screen or spray printing. **B** Schematic illustration of extrusion and inkjet printing of additive-free MXene ink. Reprinted with permission from Ref. [135]. **C** Schematic of the **a** screen printing and **c** spray printing of PVA/MWCNT inks to prepare the piezoresistive sensor (**b**). **d**–**e** show a 2% strain of the screen- and spray-printed sensors by the piezoresistive measurements for 10 loading–unloading mechanical cycles, respectively; **D** 3D printing of polylactic acid/reduced graphite oxide flexible circuits by melt extrusion layer by layer. Reprinted with permission from Ref. [137]

Inkjet printing/extrusion printing/screen printing are direct ink writing method of materials, which offers a promising strategy for scalable production of smart devices manufacturing with a high degree of pattern and geometry flexibility [132]. Inkjet printing/extrusion printing/screen printing refers to the process that melt or solution ink is jetting or extruded through the nozzle and drops onto substrate under surface tension, then solvent evaporate to form complex patterns. A schematic of different printing methods is shown in Fig. 8A. In this process, a challenge step is the preparation of inks, which require functional inks with suitable fluidic properties, in particular surface tension and viscosity. Many studies develop FPCs from inorganic fillers, such as CNTs, graphene, MXene and metal nanoparticle/nanowires or its alloy, and organic polymer, including epoxy, TPU, PDMS, PVA and polyvinylpyrrolidone (PVP), etc. [133]. For instance, Juntunen et al. reported an inkjet print graphene film with outstanding thermoelectric properties through dispersing graphene in isopropyl alcohol (IPA) with the aid of PVP [9]. In this system, PVP was used as dispersant and viscosity regulator to adjust the ink stability. During preparing water-based solvent for inject printing, it is difficult to formulate and stabilize inks due to the low viscosity and weak interfacial interaction with substrate. Besides, the coffee-ring effect (the phenomenon of droplet edge gathering) would appear during solvent evaporation. Vural and coworkers employed MXene and proteins in aqueous solution for inkjet printing and studied its EMI shielding efficiency [134]. The binder molecule proteins can form sequence controlled assemblies with hydrogen bonding MXene crystals when high-viscosity solution of (dimethylsulfoxide (DMSO) and aqueous) and aqueous evaporates on heated substrates. Hence, the role of protein is to promote MXene dispersion and adhesion between dispersion and substrate. However, the residues of surfactants mixed in ink usually bring problems to the subsequent treatment. Zhang et al. demonstrated two types inks by MXene without additive and designed for inkjet printing and extrusion printing, as shown in Fig. 8B [135]. This additive-free MXene ink exhibits general protocols, even ohmic resistors can be inkjet-printed. Gonçalves et al. [136] reported a water-based printable piezoresistive sensors by PVA filled with MWCNT, and two printing technologies, spray printing and screen printing were used in sensor printing (Fig. 8C a–c). The spray and screen printing sensors show a similar behavior and *GF* (Fig. 8C d–e) and the piezoresistive response for 10 loading–unloading cycles up to 2% strain, showing that the piezoresistive response film prepared by this ink has high universality.

As for FDM technique, the melt semi-liquid filaments are extruded layer by layer by the nozzle and then the layers are fused together and then solidify into final parts. For example, Zhang et al. printed reduced graphene oxide/polylactic acid (RGO/PLA) by dispersing RGO in PLA following melt extrusion layer by layer (Fig. 8D) [137]. This printed 3D bulk can be used as flexible circuits and printed complex structure. A number of reviews on 3D printing can be found elsewhere [132, 138]. However, there are still some limitation in 3D printing. Firstly, the surface tension and viscosity are the key factors in making printing inks, which heavily depends on the printability and stability. Second, the use of additives (such as surfactants) makes the inks more printable in most printable inks, but how to remove these additives is an urgent issue to be solved. General follow-up processing steps such as chemical treatment or thermal annealing make it more complex for the device manufacturing process. Finally, these layer-by-layer stacking approaches can limit the geometries of devices and fine resolution. Overall, overcoming these limitations potentially could result in wide range of applications.

#### (S17) Curing

Curing is a process employed by cross-linking the functional groups on the polymer to achieve toughening or hardening, which is essential on the path to prepare thermosetting polymers and their composites [139]. In the preparation of FPCs, functional fillers are generally mixed with prepolymers, and then cross-linked polymer network is solidified and formed by curing agent so as to obtain a specific network fixed in polymer. When using the method of thermal curing, solution mixing is often used to prepare a mixed system of filler and polymer or accompanied by hot pressing or other post-treatment methods. Take the most frequently used epoxy resin as an example, no matter it is simply mixing epoxy with filler and then curing or more complex strategy through film casting-filtration-hot pressing-thermal curing, curing method is often used together with other methods [94, 140].

#### (S18) Others

Besides the processing methods summarized above, there are some others processing methods for the preparation of FPCs, such as a co-coagulation method [141, 142], flocculation method [143], solvent post-treatment [144], filler sedimentation [145], vulcanization [146], roller compression [147], steam treatment [93] and light irradiation [148]. In addition, some simple means through attachment of filler and matrix could be used to prepare conductive SSPCs [149, 150]. These methods are not universally applicable during secondary processing of FPCs, but they are still effective strategies to impact on the structure, morphology and final performance.

## Selection and Combination of Preparation Tools

**Fig. 9:**
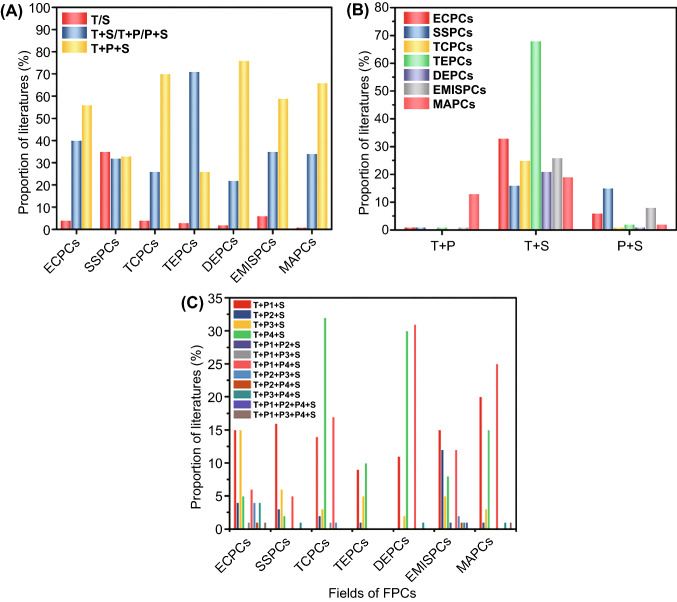
Proportion of literature for **A** single-type of tools, dual-type of tools, and ternary-type of tools in 100 literature of each field of FPCs, **B** different dual type of tools in 100 literature of each field of FPCs, **C** different ternary type of tools in 100 literature of each field of FPCs. Abbreviations: ECPCs (electrically conductive polymer composites); SSPCs (strain/pressure sensing polymer composites); TCPCs (thermally conductive polymer composites); TEPCs (thermoelectric polymer composites); DEPCs (dielectric polymer composites); EMISPCs (electromagnetic interference shielding polymer composites); MAPCs (microwave absorbing polymer composite)

For practical applications, processing method is the decisive factor to determine the structure and properties of FPCs in addition to the choice of filler and matrix. Based on the above-mentioned description of different processing tools, it is not difficult to find peculiarities of T, P and S. Thereinto, *T*-type tools represent the mixing mode of polymer matrices and fillers, most of *S*-type tools represent the approach to mold the final products, and *P*-type tools as additionally optional tools were generally utilized to control morphology of filler network and modify the interface between polymer and filler, respectively. During the preparation of FPCs, different tool combinations are selected based on the characteristics of respective research field and the requirements of morphology and performance. We summarized 100 representative literature from recent ten years for each type of FPCs, and the selection and combination of processing tools was visualized by a diagram as shown in Fig. 9. Among these studies, it is found that one functional polymer composite was able to be fabricated by only one of processing types (namely single-type of tools), combination of any two processing types (namely dual-type of tools, containing T + P, P + S, or T + S combinations) or combination of all three processing types (namely T + P + S combination). It is worth to note that non-functional polymer composites within the above-mentioned literature were prepared by only P-type tools, attributing to their intrinsic peculiarities. Moreover, different selection and combination of processing tools lead to the corresponding special structure within FPCs, and the selection and combination of processing tools diverse for different kinds of FPCs. The detailed description and summary were presented below.

### Single-type of Tools

Single-type tools mean that only one processing type from the “Toolbox” is used to manufacture given FPCs. According to summarization, single-type tools often indicate only S-type tools or merely prepared by only *T*-type tools. There is one literature wherein composites for MAPCs application with core–shell nanostructure fabricated by in situ polymerization (T2) [151]. For only *S*-type tools, common strategies to prepare FPCs are: neat polymer film, fiber, yarn or mat were firstly obtained, followed by coated, printed, injected or deposited with filler [152, 153]; or array, fabric, sponge or film of filler were fabricated at first and then combined with polymer through impregnation, infiltration, coating and attachment or LbL assembly was adopted through alternating casting, coating or filtration [13, 154].

**Fig. 10:**
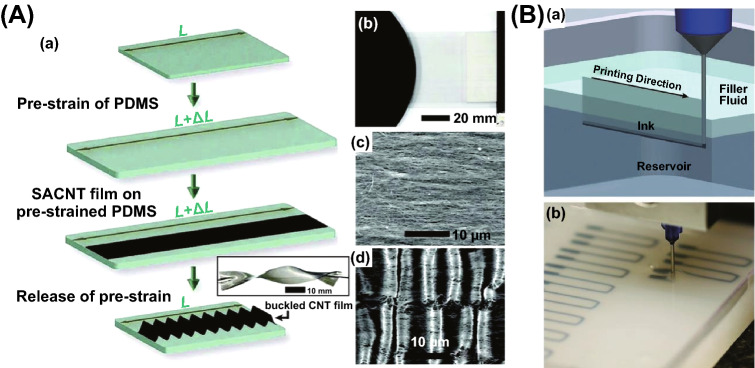
**A** Schematic illustration for preparing polydimethylsiloxane/super-aligned carbon nanotube (PDMS/SACNT) composites films (**a**), the photograph of SACNT film (**b**), SEM images of SACNT film (**c**) and the buckled SACNT structures on a PDMS film after releasing pre-strain (**d**). **B** Sketch of the embedded 3D printing process with printing a conductive ink within an uncured elastomeric reservoir capped by filler fluid (**a**) and for preparing a planar array of strain sensors (**b**). Reprinted with permission from Ref. [155]

For example, Yu and coworkers obtained wavy buckled super-aligned CNT (SACNT) films by pre-straining PDMS substrate and release (S9) to fabricate stretchable conductors as shown in Fig. 10A a [101]. This curved structure composites exhibit high stretchability and durability. For LbL assembly strategy, PVA/MXene multilayered films with excellent EMI performance were prepared through alternating multilayered casting, which exhibit a maximum EMI shielding effectiveness (SE) of 44.4 dB and a specific EMI SE (SSE_t_) of 9343 dB cm^2^ g^−1^ due to the unique alternating multilayered structure and high electrical conductivity [13]. Moreover, 3D printing is another strategy to direct fabricate FPCs with only S-type tools. As shown in Fig. 10B, a conductive ink was printed into an uncured elastomeric reservoir through embedded 3D printing (S16) process [155].

In summary, single-type of tools includes only *S*-type tools that are focused on fabricating FPCs with special structure, e.g., multilayered structure, aligned filler network structure, three-dimensional interconnected filler network structure, and hierarchical structure.

### Dual-type of Tools

Dual type of tools evidently means that two processing types were selected and combined for the preparation of FPCs, and there are three combinations: T + P, T + S and P + S. It is worth noting that generally T + S combination is the major dual type of tools to fabricate FPCs, while T + P combination was less adopted; during the fabrication of TC and DE, T + P combination was not adopted and almost all of TC and DE materials were prepared by T + S combination; three types of dual-type tools were most utilized in the fields of CPCs, MA, EMI and TE. The detail of each dual-type tools will be discussed regarding their process tools-structure–property relationship as follows.

#### *T* + *P Combination*

**Fig. 11:**
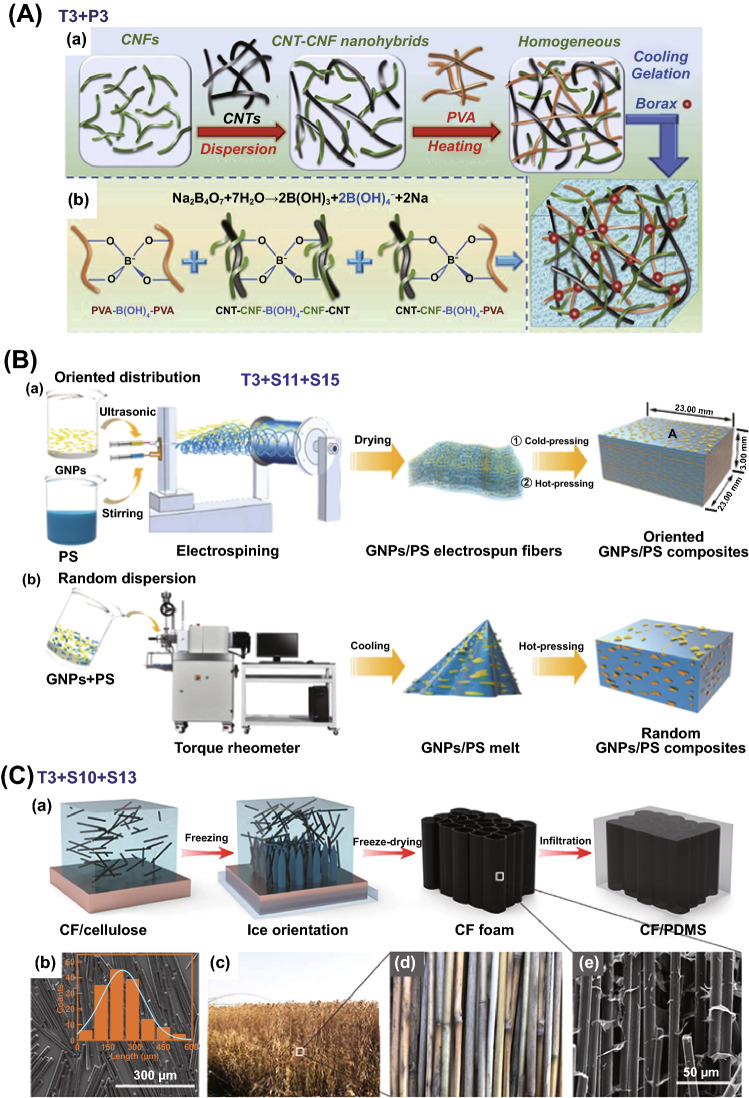
**A** Schematic illustration of the preparation procedure of carbon nanotube/cellulose nanofiller/polyvinyl alcohol (CNT-CNF/PVA) composite gels. Reprinted with permission from Ref. [156]. **B** Schematic presentation of graphite nanoplatelets/polystyrene (GNPs/PS) composites with interconnection oriented GNPs network (**b**). Reprinted with permission from Ref. [169]; **C** Preparation of carbon fibers/ polydimethylsiloxane (CF/PDMS) composites to obtain vertical CF foam microstructure. Reprinted with permission from Ref. [170]

In this part, three types of structures were fabricated without S tools: homogenous structure, multilayered structure and core–shell nanostructure. Homogenous structure tends to be often achieved in the field of ECPCs, EMISPCs and SSPCs. For example, Han and coworkers prepared self-healing CNT-CNF (cellulose nanofiller)/PVA electro-conductive hydrogels through solution mixing (T3) of CNT and CNF to obtain aqueous suspension followed by adding PVA and borax (P3), as shown in Fig. 11A [156], which illustrate a maximum electrical conductivity up to 10 S cm^−1^. Meanwhile, multilayered structure was obtained by in situ electrochemical polymerization (T2) of polyaniline (PANi) onto the surface of acid-treated SWCNT (P4) buckypaper, and then PANi/SWCNTs composite TE films with bilayer structure was obtained, achieving a maximum power factor of 6.5 μW m^−1^ K^−2^ when the electrodeposited cycles at 75 [157].

Furthermore, core–shell nanostructure could be constructed for MAPCs via a similar in situ polymerization of monomer (T2) on the surface of one filler or hybrid fillers. It is worth to note that hybrid fillers and in situ polymerization of the monomer of conductive polymers, e.g., PEDOT, PANi, PPy, are preferred [16, 158, 159]. Such combination of processing tools brought out enhanced interfacial polarization which helped in improving EM absorption abilities. For instance, SiC whiskers-graphite nanosheet/PPy materials with core–shell nanostructure were prepared through in situ polymerization of pyrrole on the surface of hybrid SiC-graphite nanosheets (GNs) fillers (P1), resulting in that minimum reflection loss (RL_min_) value of − 64.2 dB [160] and 7.9 GHz bandwidth. Similar sandwich nanostructure also was obtained thorough in situ polymerization [161]. Generally, T + P combination mainly includes the combination of in situ polymerization (T2), solution blending (T3) or T2 + T3 with hybrid fillers (P1), blends (P3), filler surface modification (P4), P1 + P4 or P3 + P4, leading to three types of structures including homogenous structure, multilayered structure and core–shell nanostructure.

#### *T* + *S Combination*

T + S combination is the frequently used dual-type tools in FPCs field, which endows these FPCs with more plentiful filler network structure, e.g., random filler dispersion structure, oriented filler network structure, and “brick–mortar” multilayered structure. Herein, T1 + S, T3 + S and T4 + S are discussed firstly as follows. Directly mixing polymer matrices and fillers to develop solid FPCs with fillers randomly dispersed in polymer components is the general and most important strategy of T + S combination, which is involved in all seven fields of FPCs. Random filler dispersion structure is common and important structure. Meanwhile, the homogenous filler dispersion within the polymer matrix is generally the target. For example, the combination of solution blending (T3) and film casting (S8) allows the preparation of PVDF/Ta_4_SiTe_4_ composites with Ta_4_SiTe_4_ whiskers homogeneously dispersed in PVDF matrix for thermoelectric application [162]. The PVDF/Ta_4_SiTe_4_ composites demonstrate a maximum power factor value of 1060 mW m^−1^ K^−2^ at about 220 K in the in-plane direction.

Different from the random filler dispersion, aligned, oriented or ordered fillers network as another important filler network structure could be constructed via T + S combination, wherein magnetic/electric field (S6) [163, 164], hot drawing/melt spinning (S4) [165, 166], injection molding (S5) [167], electrospinning (S11) [6] or freeze drying (S13) [118] combined with corresponding melt blending (T1) or solution blending (T3), namely combination of T1 or T3 with S4, S6, S5, S11 or S13, that have been proved to form aligned fillers network within polymer matrix. Specifically, aligned Co nanowires network within PVDF matrix was prepared via combination of solution blending (T3), drop casting (S7) and magnetic field (S6). This contributes to obviously increase electrical conductivity in comparison with that random filler dispersion. Such composites illustrate an impressive power factor value of 523 μW m^−1^ K^−2^ at 320 K [89]. In the field of ECPCs, Zhu et al. fabricated conductive PVA/GN nanocomposites based on solution blending (T3), film casting (S8) and hot drawing (S4) successively. Due to the hot drawing treatment on PVA/graphene composites, aligned graphene conductive network was observed and a superior conductivity value of 25 S m^−1^ at 6.25 wt% reduced graphene oxide with slightly oxygen content (SRGO) loading was achieved [168]. Moreover, the processing method containing solution blending (T3), electrospinning (S11) and compression (S15) could form oriented graphite nanoplatelets (GNPs) network (Fig. 11B) within PS matrix and illustrate significantly enhanced EMI performance [169]. In contrast, GNPs/PS composites with oriented GNPs network demonstrate much higher total EMI SE (SE_*T*_) (− 33 dB) than that of GNPs/PS composites with random GNPs distribution (− 16 dB) at the same GNPs content of 35 wt%. It is worth to mention that PDMS/CFs composites were prepared as follows: solution blending cellulose and CFs, immersing in the liquid nitrogen to freeze drying to obtain foam, then PDMS was pour casting in CFs, successively (Fig. 11C a). During freeze in liquid nitrogen, ice crystals randomly and rapidly grew from bottom and CFs were squeezed by ice crystals, reducing to CFs arranging in parallel with each other guided by ice crystals. Therefore, CFs foam with phragmites-like communis-oriented microstructure is obtained as shown in Fig. 11C b–e. Owing to this special structure, the through-plane thermal conductivity of 6.04 W m^−1^ K^−1^ is observed at 12.8 vol% loading of CFs [170].

In this pattern (T + S), the use of combination of *T*-type tools and a variety of *S*-type tools to build some special network structure is a preferred choice, such as solution or melt blending followed by freeze drying (S13) or foaming (S1) or LbL assembly (S14) [171–173]. For example, Zhao et al. utilized three-step approach including solution blending (T3), compression molding (S15) and batch foaming (S1) to prepare PVDF/CNT nanocomposites with wideband microwave absorption properties [173].

For the combination of T2 and S-type tool to fabricate FPCs, it was mainly adopted in TEPCs field. For example, PEDOT/CNT thermoelectric composites with core–shell nanostructure was fabricated by in situ polymerization (T2) and vacuum filtration (S10). The strong interfacial interaction and carrier transport caused by in situ polymerization resulted in a rather high power factor of ~ 157 MW m^−1^ K^−2^ [174]. In addition, in situ polymerization of aniline onto the surface of various fillers, e.g., SWCNTs [174], MWCNTs [175] or GO [176], could lead to ordered PANi chain structure; this ordered PANi chain structure could facilitate carrier mobility and therefore enhanced electrical conductivity, Seebeck coefficient and power factor are obtained in comparison with pure PANi.

It can be seen from the above discussion that FPCs fabricated by T + S combination are more focused on modulating filler dispersion state within polymer matrix and interfacial interaction between filler and polymer. Meanwhile, more combination of processing tools can be adopted for preparing FPCs with optimized structure and enhanced corresponding performance due to the introduction of *T*-type tools.

#### P + S Combination

P + S combination could endow FPCs with more plentiful structure originating from both *P*-type and *S*-type tools. Most of the combination of *P*-type tools and *S*-type tools includes four patterns: P2 + S, P1 + S, P4 + S or the other combination P1 + P4 + S. We will discuss examples based on the above summarization of literature in more detail as follows.

**Fig. 12:**
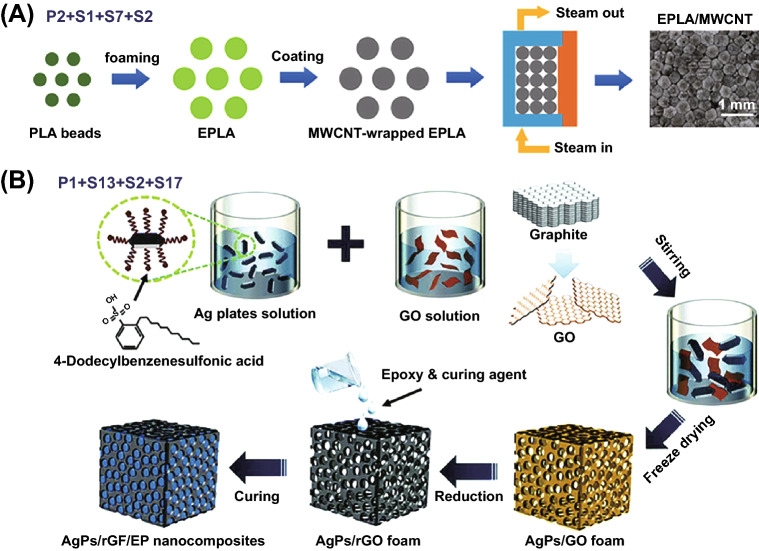
**A** Schematic illustration of the fabrication process of polylactic acid (PLA)/MWCNT composite foam with segregated conductive CNTs network. Reprinted with permission from Ref. [177]. **B** Sketch of the fabrication of the AgPs/rGO foam/epoxy composite materials. Reprinted with permission from Ref. [178]

For P2 + S, porous PLA/MWCNT composites with segregated conductive networks (P2) for EMI applications were prepared by obtaining expanded PLA (EPLA) beads after foaming PLA pellets, immersing in MWCNTs solution to wrap a layer of MWCNTs (S7) and then sintering MWCNT-wrapped EPLA to obtain PLA/MWCNTs nanocomposite foams (S2), as shown in Fig. 12A. The foams illustrate an inspiring EMI SE of 1010 dB cm^3^ g^−1^, owing to the unique architecture of fine microporous matrix containing conductive MWCNT networks, making it difficult for EM waves to escape [177]; For P1 + S in the fields of both ECPCs and EMI, interconnected spherical hollow conductive networks were constructed within silver platelets (AgPs)/RGO foam/epoxy composites by processing procedure as follows: firstly mixing GO and AgPs suspension (P1) followed by freeze-drying the mixing suspension (S13) to form a 3D framework, then thermal annealing the framework (S2 tool) for the reduction of GO to obtain 3D AgPs/RGO framework, at last pouring epoxy resin into this framework (S10) and curing (S17) as demonstrated in Fig. 12B [178]; As to P4 + S, take TEPCs field as an example, PEDOT:PSS/Bi_2_Te_3_ composites fabricated via treating Bi_2_Te_3_ particles with HCl (P4), followed by dip coating (S7) Bi_2_Te_3_ dispersion and PEDOT:PSS solution in sequence [179], wherein the treatment by HCl could remove the oxidized layers on the Bi_2_Te_3_ particles surface to achieve a more electrically conductive interface between PEDOT and Bi_2_Te_3_, and therefore obtaining both higher electrical conductivity and Seebeck coefficient; for P1 + P4 + S, Kim et al. reported thermally conductive GO/MWCNT/epoxy composites by firstly preparing GO nanosheet and surface-modifying MWCNT with carboxylic acid functional groups (P4), then mixing GO and MWCNT suspension (P1 tool) and form a GO/MWCNT cake using vacuum-assisted filtration. It is followed by wetting the cake with epoxy resin (S10) and curing (S17). Within these GO/MWCNT/epoxy composites, MWCNTs act as interconnectors between GO and heat conductive bridges among the 3D microparticles due to their high aspect ratio, leading to over 140% of maximum enhancement ratio for thermal conductivity compared with GO/epoxy composites without MWCNTs [50]. In summary, the introduction of *P*-type tools aimed at modulating filler network structure by hybrid fillers, polymer blends, volume excluding particles as well as improving interface between filler and polymer by filler surface modification. *P*-type tools are also considered to be materials selections before FPCs are processed into desired shape and structure.

### T + P + S Combination

T + P + S combination has more multi-variant combination modes of processing tools in practice and generally more plentiful microstructures within FPCs. They are the most frequently used combinations in the literature. *S*-type tools could endow the composites with desired structure, i.e., film, foam, aerogel, multilayered structure, or some special filler network structure, e.g., oriented filler structure, segregated filler network, 3D interconnected filler network. Compared with T + S combination, introducing hybrid fillers (P1), volume excluding particle (P2), polymer blends (P3) and filler surface modification (P4) could further improve the dispersion state of fillers within polymer matrix, strengthen the interfacial interaction between filler and polymer and modulate filler network structure. Moreover, in contrast to P + S combination, the issue of T + P + S concerning on the dispersion state and network structure of fillers in the polymer matrix as well as the interface between filler and polymer emerges. Here, the discussion on T + P + S combination was conducted based on comparison with T + S combination and attention was mainly paid on the *P*-type tools.

**Fig. 13:**
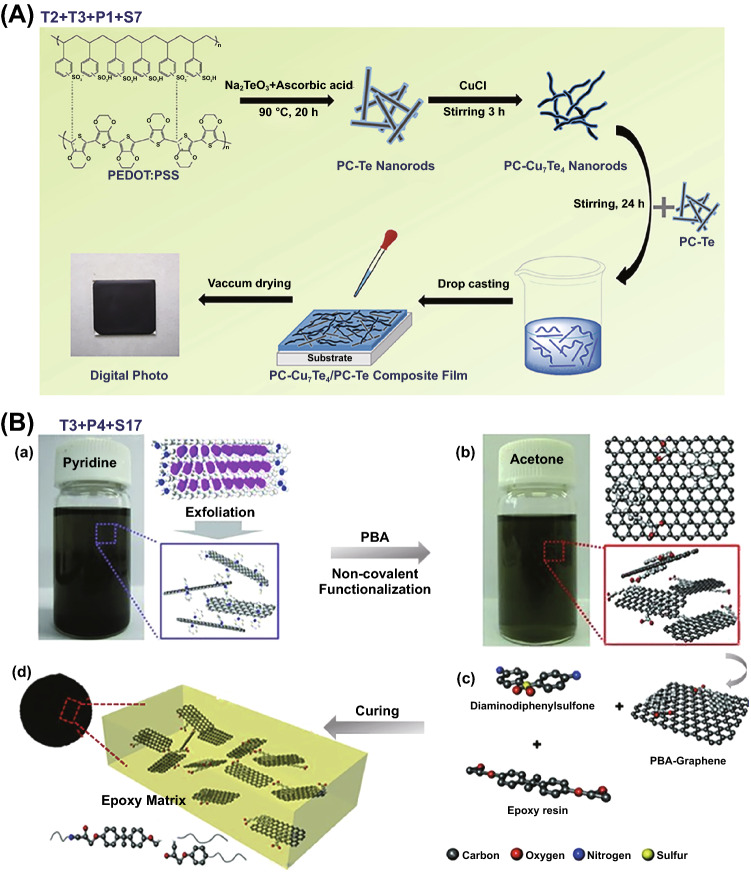
**A** Schematic illustration of the fabrication process for polycarbonate (PC)/Te/Cu_7_Te_4_ thermoelectric composites. Reprinted with permission from Ref. [183]. **B** Schematic plot of the fabrication for epoxy/f-GFs composites. Reprinted with permission from Ref. [186]

In this part, there are numerous kinds of combination of processing tools selected from “Toolbox” and the use of combination of processing tools among seven fields of FPCs is different. Comparing T + P1 + S combination [180, 181] with T + S combination, P1 could bring out hybrid fillers network structure and synergistic effect between different fillers, which is helpful to improve the corresponding performance. For example, Voit et al. fabricated PVDF/CNTs/CB composites by melt blending (T1) three components in a twin-screw microcompounder (S3) and then hot compression (S15). CNTs and CB formed string-like conductive network structures composed of CNT-CB-CNT block arrangement within PVDF matrix, therefore resulting in higher Δ*R*/*R*_0_ than that of composites consisting of only CNTs [182]. In the field of TE, in situ synthesis method (T2), solution mixing (T3) and drop casting (S7) were used to prepare PEDOT:PSS PC/Te/Cu_7_Te_4_ (P1) composites, which demonstrate a maximum power factor of 112.3 μW m^−1^ K^−2^ at temperature of 380 K (Fig. 13A). Such performance is ascribed to the synergetic effect of PC/Te and PC/Cu_7_Te_4_ nanorods as well as the double-carrier filtering effect at the hetero-interfaces of PC/Te and PC/Cu_7_Te_4_ [183].

Comparing T + P4 + S with T + S combination, filler surface modification (P4) usually can improve the dispersion of fillers and interfacial interaction between polymers and fillers [184, 185]. For instance, epoxy/GN composites containing non-covalent functionalized non-oxidized graphene flakes were prepared by the procedures composed of non-covalent surface functionalizing graphene flakes (GFs) with pyrenebutyric acid (PBA) (P4) to obtain f-GFs, solution blending f-GFs with epoxy (T3) and curing (S17) in turn (Fig. 13B). Herein, the presence of interaction of carboxylic group with epoxy matrix enhances the interfacial adhesion and dispersion of GFs within epoxy, leading to the highest thermal conductivity of 1.53 W m^−1^ K^−1^ at 10 wt% loading of f-GFs in epoxy compared with other fillers: CB, graphite, MWNT and GO [186].

**Fig. 14:**
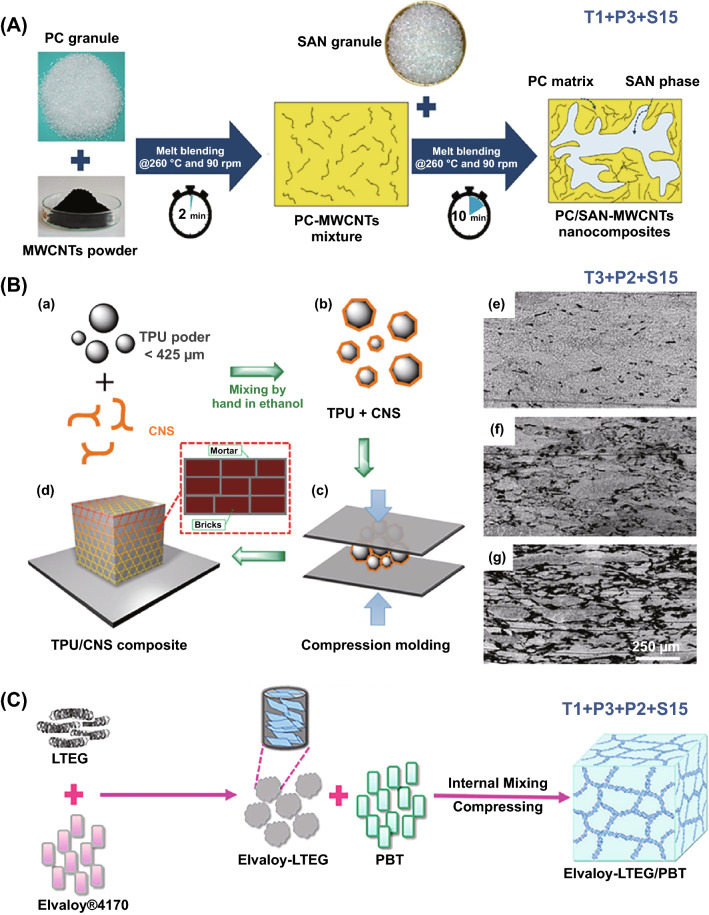
**A** Schematic plot of the fabrication for PC/SAN(polycarbonate/PS-co-acrylonitrile)/MWCNTs composites. Reprinted with permission from Ref. [192]. **B** Sketch of the preparation procedure and characterization of thermoplastic polyurethane carbon nanostructures (TPU/CNS) composites with segregated CNS network structure. Reprinted with permission from Ref. [199]. **C** Schematic of the preparation procedure for Elvaloy@4170 low-temperature expandable graphite/poly(butylene terephthalate) (LTEG/PBT) composites with segregated network. Reprinted with permission from Ref. [212]

Furthermore, in comparison with T + S combination, introducing polymer blends (P3) is an unique strategy in this part to construct peculiar microstructures, especially in the field of CPCs, where T1 + P3 + S [187, 188], T3 + P3 + S [113, 189, 190], or T1 + T3 + P3 + S [191] was often used to fabricate composites with selective distributed fillers through polymer blends with different morphologies. For example, Bizhani and coworkers reported PC/PS-co-acrylonitrile (SAN)/MWCNTs composites fabricated by T1 + P3 + S15 strategy: first melt blending PC and MWCNTs and then melting blending PC/MWCNTs composites with SAN followed by hot compressing (Fig. 14A) [192]. In PC/SAN/MWCNTs composites, PS/SAN illustrates co-continuous morphology, and MWCNTs are preferentially localized in PC phase, leading to an electrical double percolation threshold around 0.32 wt% of MWCNTs and a maximum EMI SE value of − 25 ~ − 29 dB containing only 1 wt% MWCNTs at a thickness of 10 mm due to the special conductive pathway. Similar co-continuous morphologies of polymer blends were also constructed in other studies to modulate conductive networks [193]. Besides co-continuous morphologies, other morphologies of polymer blends, e.g., polymer blends with tri-continuous morphology [57], immiscible polymer blends [194] were also adopted to control the filler network.

Moreover, as shown in Fig. 9C, T + P4 + S was preferred in the field of TCPCs and DEPCs in comparison with other fields. This indicates that filler surface modification was more frequently used to prepare TCPCs and DEPCs. For TCPCs, filler surface modification involved diverse methods, such as grafting [195], group decoration [196], inorganic functionalization [197] and organic surface modification [92]. In DEPCs field, filler surface modification was mainly applied to form core–shell structure with filler as core and polymer as shell to homogeneously disperse fillers within polymer and strengthen the interfacial interaction. For TCPCs, homogeneous dispersion of fillers and enhanced interfacial interaction between filler and polymer lead to reduced thermal resistance and phonon scattering and thus improve thermal conductivity, while for DEPCs, homogeneous distribution of filler leads to reduced concentration of electric field in the polymer matrix; thus, breakdown strength can be enhanced. Meanwhile, good dispersion of filler and enhanced interfacial interaction between filler and polymer result in high dielectric constant and low dielectric loss [198].

P2 is a special primary processing tool to obtain segregated network structure that almost only emerges in T + P + S combination. For example, in the field of SSPCs, stretchable strain sensors with robust segregated network structure were prepared by Sang et al. based on TPU and carbon nanostructures (CNS) via grinding mixing TPU power and CNS (T3), followed by directly compression molding (S15) after drying (Fig. 14B a–d). In such composites, TPU phase acts as polymer matrix as well as volume excluding particle (P2) and CNS trapped at the interfaces of TPU powers, forming interconnected conductive networks throughout the composites, as demonstrated in Fig. 14B e–g. These strain sensors demonstrate a high gauge factor of 6861 at strain *ε* = 660% with 0. 7 wt% filler and a few orders of magnitude higher electrical conductivity, much higher elongation at break than that of TPU/CNS composites at the same filler content fabricated by melt mixing [199]. Besides, the combinations of T2 + P2 + S or T3 + P2 + S could also be applied to construct segregated network structure [200].

In contrast to other combination of T + P + S containing two kinds of T-type tools, T + P1 + P4 + S was more preferred for fabricating DEPCs and MAPCs. For T + P + S combination containing P1 + P4, a strategy containing fabricating core–shell hybrid fillers and then blending with polymer was a vital processing method to obtain composites with excellent dielectric performance [201], wherein most of the core–shell hybrid fillers had insulting shells, e.g., Al_2_O_3_ shells, ZnO shells, GO shells [202, 203], and these insulting shells could suppress formation of conductive pathways and carrier migration and therefore reduce the leakage current as well as dielectric loss. For example, PVDF/core–shell CaCu_3_Ti_4_O_12_@Al_2_O_3_ (CCTO@Al_2_O_3_) nanofibers composites was fabricated via the following procedure: (i) core–shell CCTO@Al_2_O_3_ nanofibers (P1, P4) were fabricated via coaxial electrospinning and then high-temperature calcination; (ii) CCTO@Al_2_O_3_ nanofibers were surface modified with PDA (T4); (iii) PVDF/CCTO@Al_2_O_3_ nanofibers composites were prepared by solution blending modified nanofibers with PVDF (T3), film casting (S8) and thermal annealing (S2) [204]. The insulating layer of Al_2_O_3_ could reduce the charge accumulation and thus decrease the leakage current density and dielectric loss. In addition, Al_2_O_3_ layer also acts as buffer layer and reduces the dielectric constant difference between PVDF and CCTO, resulting in a higher electric breakdown strength in comparison with that of PVDF/CCTO composites at the same filler content. Apart from the insulating layer, surface decoration of Ag [205], Fe_3_O_4_ [206] was also used to fabricate core–shell hybrid fillers.

Moreover, there are still many studies wherein similar processing methods or others containing T + P1 + P4 + S combination were used to fabricate other types of FPCs [207, 208]. Additionally, hybrid fillers (P1) or filler surface modification (P4) was introduced to combine with T + P3 for the preparation of FPCs [209, 210]. The combination of polymer blends (P3) and volume excluding particle (P2) is another effective strategy to fabricate FPCs with segregated filler network [211]. For instance, our group added low-temperature expandable graphite (LTEG) into a commercial impact modifier (Elvaloy4170) and then coated onto poly(butylene terephthalate) (PBT) particles to fabricate PBT/Elvaloy4170/ LTEG thermally conductive composites by combining two-step internal mixing (T1) at preset temperature and hot compression (S15) (Fig. 14C). In such composites, a continuous segregated filler network is constructed by continuous Elvaloy4170-LTEG phase and segregated PBT particles phase (equivalent to volume excluding particles (P2, P3). These composites exhibit a maximum thermal conductivity up to 17.8 W m^−1^ K^−1^, which was significantly prior to that of composites made by directly melt mixing three components at the same filler content. Meanwhile, significant effect of PBT particles size, namely segregated phase size, on the thermal conductivity of PBT/Elvaloy4170/LTEG was also reported [212]. Besides, some more complicated combinations, such as T + P1 + P2 + P4 + S or T + P1 + P3 + P4 + S, were also applied to prepare ECPCs, EMISPCs or MAPCs. In the field of ECPCs, solution blending (T3), hybrid fillers (P1), blends (P3), filler surface modification (P4), freeze drying (S13) and hot pressing (S15) were applied for preparing styrene-butadiene rubber (SBR)/PVP/RGO/CNT composites, wherein PVP acts as dispersing agent to improve the dispersibility of RGO and CNTs hybrid fillers. Due to the sandwich structure of RGO-CNTs hybrid fillers, restacking of RGO and agglomeration of CNTs were prevented, and thus, a high electrical conductivity was obtained compared with SBR/PVP/CNT at the same filler content [213]. This T + P + S combination with three kinds of P-type tools was merely adopted to fabricate FPCs, which is attributed the tedious processing procedure and relative high cost.

In conclusion, there are numerous selections of processing tool for fabricating FPCs. However, the selection and combination of processing tools varies obviously among seven fields of FPCs as discussed above. On the one hand, the combination of processing tools for seven types of FPCs depends on different mechanisms underlying the structure–property relationship in each field; on the other hand, owing to similarities, e.g., containing polymer matrix and filler, involving filler network and interfacial interaction, different fields of FPCs could learn from each other.

## Summary and Future Prospects

FPCs are composed of fillers and polymer matrices, while the various functionalities and properties of different composites can vary greatly. In the past decade, a variety of FPCs have been synthesized and explosive progress has been achieved for different functionalities, including electrical conductivity, strain sensing ability, thermal conductivity, dielectric properties, electromagnetic shielding (EMI), microwave absorption and thermoelectric property. These functional properties are largely determined by filler, polymer, network morphology and interfacial interaction. All of these could be considered as important components of processing method when choosing processing strategy for certain type of FPCs. In this review, the processing strategies for FPCs are summarized into a “Toolbox” to cover recent progress as well as guide further research. The relationship of processing method–structure–property is discussed and the selection and combination of tools in processing from 700 studies among different FPCs are also analyzed.

In the “Toolbox” system, the mechanism in the field of FPCs is the cornerstones for the preparation of FPCs. Under its operation, we have a more directional choice of processing methods and meet the design requirements of FPCs better. The “Toolbox” consists processing type, primary processing tools and secondary processing tools for better understanding of the processing methods. The processing methods in the “Toolbox” are tools available to prepare FPCs, just like hammer or screwdriver in the engineering toolbox. The establishment of such “Toolbox” not only categorizes and summarizes the progress of current processing methods, but also is expected to guide the design of FPCs in the future. Based on the information given by the “Toolbox,” the processing methods from different fields can learn from each other. Preparation methods commonly used in a specific field may bring fresh ideas for the preparation of FPCs in another field. Furthermore, new methods could be created by combining tools from “Toolbox” with different sequence as well as quantity. The combination of tools is not randomly done, it is understood that these tools are combined together with certain suitability due to various issues.

**Fig. 15:**
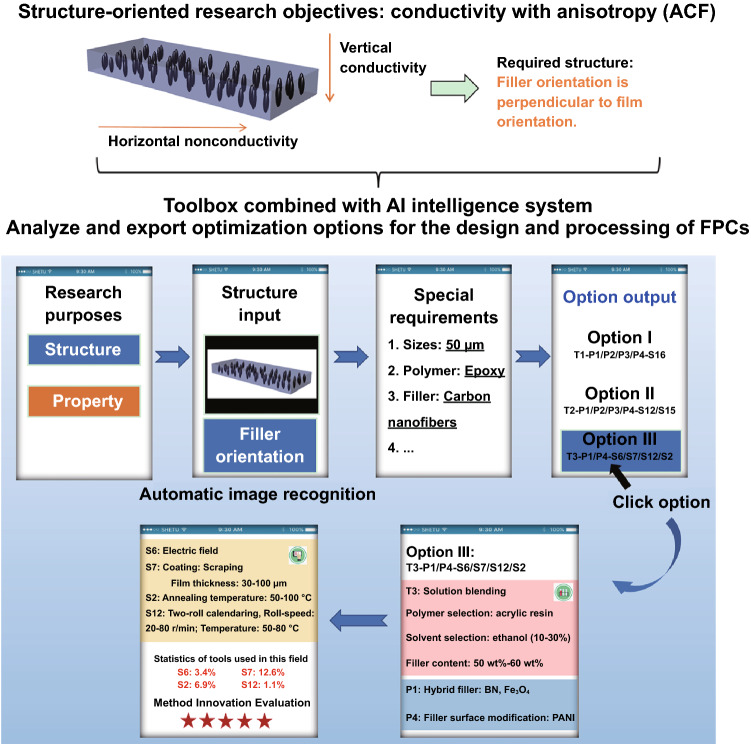
Demonstration of the application for morphological control “Toolbox” in combination with artificial intelligence

The future application trend of such “Toolbox” is prospected in this review. It is speculated that prediction on the processing tools applicable for certain FPCs could be made by computer software once “big data” on processing–structure–properties relationships of various FPCs is collected and summarized. By combining with “artificial intelligence” (AI) computer program, the concept of “Toolbox” program could be used to collect and summarize the overall materials database, and then, possible processing routes for given structure or targeted properties could be predicted and designed. It is thought that various pieces of information on certain study should be collected for the database: (i) the filler network structure realized by various processing method; (ii) the degree of improvement in certain functionality due to the utilization of different processing method; (iii) the type of polymers and fillers used for respective processing methods; (iv) the processing condition often used for certain type of processing methods. As shown in Fig. 15, structure purpose is used as an example to demonstrate the application for morphological control “Toolbox” in combination with artificial intelligence.

The design of materials with anisotropic conductivity is used as an example, where vertical conductivity is preferred. Firstly, we click to enter the intelligent front-end interface, select the design FPCs for the structure purpose, next upload the structure of our target, and then enter the next interface to put forward some special requirements such as size, filler and polymer matrix. After calculation, the optimal solutions of option-I, option-II and option-III would be exported for our choice. All this is based on the comprehensive database of the Toolbox. Besides, based on the system of database collection, “Toolbox” could also be used for performance evaluation. In short, the establishment of the “Toolbox” database has three benefits: (i) It is the cooperation of an open sharing platform; (ii) it is to efficiently implement the research and development of new functional materials by means of self-learning ability of artificial intelligence; and (iii) it can greatly shorten the research and development cycle of FPCs. Therefore, such method could be used to guide the future application of FPCs to achieve outstanding performance more efficiently and effectively.

## Supplementary Information

Below is the link to the electronic supplementary material.

Supplementary file1 (PDF 289 kb)
