# Supplementary material for: “Toolbox” for the Processing of Functional Polymer Composites
Source: Nanomicro Lett. 2021 Dec 16;14:35. doi: 10.1007/s40820-021-00774-5 (PMC8677876; doi:10.1007/s40820-021-00774-5)
Supplement: Supplementary file 1 — Supplementary file1 (PDF 289 kb) [file 40820_2021_774_MOESM1_ESM.pdf]

Supporting Information for

# "Toolbox" for the Processing of Functional Polymer Composites

Yun Wei[1, #], Hongju Zhou[1, #], Hua Deng[1, *], Wenjing Ji[1], Ke Tian[1], Zhuyu Ma[1], Kaiyi Zhang[1], Qiang Fu[1]

[1]College of Polymer Science and Engineering, State Key Laboratory of Polymer Materials Engineering, Sichuan University, Chengdu 610065, People's Republic of China

[#] Yun Wei and Hongju Zhou contributed equally to this review article

*Corresponding author. E-mail: huadeng@scu.edu.cn (Hua Deng)

## S1 Background of Functional Polymer Composites

### S1.1 Electrically Conductive Polymer Composites (ECPCs)

ECPCs or conductive polymer composites (CPCs), comprising insulating polymer matrices and conductive filler, have found their versatile applications from public transportation, infrastructure to more specialized fields as radiation protection, antistatic etc. [S1] Due to its good processability, tunable properties and widespread applications, ECPCs is nowadays one of the most interesting and researched areas in polymer composites.

According to the final application requirements of ECPCs, the selection and choice of filler type must be carefully considered. To date, conductive nanofillers such as CNTs, carbon black (CB), graphene, metal nanowires (NWs), etc have been widely incorporated into matrices for preparing ECPCs, enabling them to be used as multifunctional materials [S2]. Nevertheless, their processability and mechanical properties are often compromised with increasing filler content [S3].

Generally, the preparation methods and protocols, which determines the network structure constructed by conductive filler, have strong impact on the final properties. Therefore, controlling the morphology of conductive network in polymer matrix have been widely studied. As shown in literatures [S4], higher filler content and better dispersion of conductive fillers do not always result in higher conductivity. A homogeneous filler dispersion is not always the best idea to achieve excellent performance of ECPC. For instance, data available in the literatures show that the percolation threshold ($P_c$) of ECPCs increases with optimizing filler dispersion status, while aggregates formed by conductive filler in the matrix can reduce $P_c$ [S5]. Nevertheless, a good dispersion of fillers is favorable for improving the mechanical properties of ECPC owing to the interfacial interaction between polymer matrix and filler is greatly improved. Based on the above issues, it can be reasonably expected that the electrical properties of ECPCs would change greatly due to processing related issues.

### S1.2 Strain Sensing Polymer Composites

Flexible and stretchable strain sensors can convert physical deformations into measurable electrical signals, which could have widespread applications such as damage detection, human motion tracking, personal healthcare monitoring, human-machine interfaces and so on [S6]. Traditional metallic or semi-conductive sensors show limited deformations i.e. less than 5% strain. Thus, they are not suitable for large-strain sensing applications. In contrast, flexible

and stretchable strain sensors are able to perceive both low and high strain levels. For majority of ECPCs with elastomers coated with conductive thin-film layers or incorporated conductive nanofillers into polymer matrices or encapsulation materials, elastomers/polymer/encapsulation materials are the stretchable component. Due to their light-weight, easy processing, flexible and stretchable, ECPCs based sensors have been developed in the last decades and turned out to be promising candidates for smart flexible strain sensors.

From previous section, it is observed that the electrical conductivity of ECPCs is determined by filler concentration, which involves percolation threshold. When an external stimulus is applied, the change of resistance signal in these conductive networks are recorded during stretching or releasing. In order to construct desirable percolating networks for strain sensing, suitable large fillers aspect ratio or synergistic effect between fillers [S7], and special phase morphology (such as segregated structure, double percolation structure, and porous structure) [S8] double percolation structure [S9], and porous structure [S10] are often required to achieve high performance strain sensitivity. In addition, a large number of processing methods are put into practice for the preparation of strain sensors, including melt-blending, solution blending, in situ polymerization, and combined with various coating (dip-coating, spray-coating, spin-coating, etc.) and others technologies [S11].

Generally, the morphology of these conductive network and interfacial interaction are the most critical issues that influence the destruction and reconstruction of conductive paths during stretching-releasing cycles, thus, producing significant influences on the sensing performance of these sensors. As a result, some complex hierarchical structures have been developed to tune the strain sensitivity of ECPCs [S12, S13]. In addition, stretchability can be further optimized by geometric engineering of conductive networks to minimize the effect of mechanical strain, such as special structures like wrinkle, double helical, buckled, wavy, coiled, serpentine pattern and spring-like [S11]. Further more, the mechanism related to the changes in tunneling distance between conductive nanofillers as well as the physical contact of these conductive network under strain helps us better explain and understand the sensitive behaviors of different types of ECPCs-based strain sensors: tunneling effect; disconnection mechanism; crack propagation mechanism [S14-S16].

## S1.3 Thermally Conductive Polymer Composites (TCPCs)

Thermal conductivity is a fundamental property for solid materials [S17]. In solids, heat can be transported by phonons, electrons or photons. In most polymers, phonons are the primary mechanism for heat conduction originated from vibration in a rigid crystal lattice. Thermal conductivity is dominated by phonons in insulators and semiconductors. As well known, due to inherent defects such as voids, impurities, polymer chain ends, and entanglements, bulk polymers have relatively low thermal conductivity within the range of 0.1-0.5W $(mK)^{-1}$, which is far below the level required by many industrial applications [S18]. Typically, in order to obtain high TC polymer-based materials, highly thermal conductive fillers are added into the polymer matrix to enhance its thermal conductivity [S19]. Thermal conductive fillers are classified into several types as follows: metallic fillers, such as Cu, Al, Ni, Ag; carbon based fillers [S20-S22], including graphite, CNT, CF, graphene, etc.; ceramic fillers [S23-S25], including metal oxide fillers such as $Al_2O_3$ and $SiO_2$, as well as non-oxide fillers such as BN, SiC, $Si_3N_4$ and AlN. The selection of these fillers is based on the requirement from different applications, such as whether electrical insulation is required, cost, processing methods, other physical properties.

In general, the key to achieve high thermally conductive composites is constructing continuous filler networks in polymer matrix. Thermal conductivity of polymer-based composites is a function of the thermal conductivities of polymer matrix and fillers, the morphology of polymer composites and the interfacial interaction between polymer matrix and fillers. Thermal conductivity of polymer matrix plays a crucial role in determining the overall thermal conductivity of polymer-based composites. The chain structure, crystallinity, crystal form and chain orientation of polymer matrix have significant influence on the thermal conductivity. Besides, it was found that a high thermal conductivity was achieved through fabricating polymer blends by engineering inter-chain interaction [S26]. As for fillers, apart from their intrinsic thermal conductivity, the main factors affecting the thermal conductivity of polymer-based composites including filler loading level, filler shape, filler size, hybrid fillers and surface treatment of fillers. Thereinto, thermal conductivity of composites nonlinearly increases with increasing volume fraction of thermal conductive filler. Generally, when the filler loading exceeds 35 vol%, a more drastic improvement in thermal conductivity could be obtained [S27].

### S1.4 Thermoelectric Polymer Composites

With the depletion of fossil fuels, renewable energy sources are essential to solve the global energy crisis. Thermoelectric (TE) materials are crucial in renewable energy conversion technologies, which enable the direct conversion between heat and electricity with a set of advantageous characteristics, such as no moving parts, no noise generation, long operating lifetimes, excellent reliability and easy maintenance [S28]. Thermoelectric materials have been widely applied in aerospace, cogeneration, refrigeration and sensors, etc. Inorganic TE materials are classic TE materials, such as $Bi_3Te_2$, $Sb_2Te$, PbTe [S29], which have attracted most of the ongoing research due to their high Seeebeck coefficient and moderate electrical conductivity. However, their applications are hindered by a number of weaknesses, including high-cost, toxicity of the elements, complicated and difficult processing methods, heavy and rigid, etc. [S30] Furthermore, flexible, durable, ecofriendly, long-lifetime and low-cost thermoelectric devices are in high demand with the emergence of fast-developing fields of wearable devices. Thereby, organic polymer thermoelectric materials have received extensive attention due to their intrinsic advantages such as flexibility, low thermal conductivity, low cost, ease of processing, low or no toxicity and light weight [S31]. These issues are beneficial for the development of flexible and portable thermoelectric modules. Conventional organic thermoelectric materials are conducting polymers like PEDOT, PANI, polythiophene (PTh), PPy, and their derivatives [S32, S33]. Due to the low thermal conductivity of thermoelectric polymeric materials (usually in the range of 0.1~0.5 W $m^{-1}$ $K^{-1}$), power factor is often employed to replace ZT for the evaluation of thermoelectric performance [S28].

For ideal thermoelectric materials, *ZT* should be $>1$ to obtain a conversion efficiency of >10% [S34]. Unfortunately, the *ZT* values of most organic thermoelectric materials are below 1 at room temperature. Thus, it is still a significant challenge to achieve high *ZT* for organic thermoelectric materials. For neat conductive polymers, their major drawback is poor electrical transport properties, which can be enhanced by doping [S35], secondary doping [S36] and altering the chain structure [S37]. Apart from that, another vital approach is to fabricate organic/inorganic hybrids wherein combining the outstanding flexibility and low thermal conductivity of organic conductive polymers and high Seebeck coefficient and electrical conductivity of inorganic counterparts [S38]. Meanwhile, the interaction between filler and polymer matrix can often yield decoupled thermoelectric parameters, and thus achieving promising thermoelectric performance compares with individual components [S39].

## S1.5 Dielectric Polymer Composites

Dielectric materials have received great attention in capacitors field with the fast development of the miniaturized and integrated electronic devices. Dielectric capacitor is a type of high-efficiency energy storage device which have ultra-high power density comparing with other energy storage devices (lithium-ion batteries, batteries and super capacitors). Recent years, considerable interests have been aroused and great efforts have been done to explore the potential of capacitors, which is urgently needed in various electronic devices [S40, S41].

Traditional dielectric materials are high temperature resistant, high strength, and high dielectric constant ceramic materials, but the disadvantages are difficult processing ability, high density, rigidity, and low energy density. Comparing with ceramic materials, polymer materials have the advantages of ease of processing, flexibility, high breakdown strength, low density, and light weight. However, because polymers are composed of long molecular chains, the ultra-long molecular chain structure reduces the polarity of the materials, resulting in rather low dielectric constant ($\varepsilon_r < 10$) [S42]. For example, the BOPP has a breakdown strength of up to 700 kV mm$^{-1}$, with a low permittivity values (2-3) [S43]. Combining the energy density formula with the characteristics of both filler and polymer, it is imperative to prepare dielectric polymer composites containing high dielecric constant fillers. Polymer matrix provides high breakdown and excellent processing property. Meanwhile, high dielectric constant ceramic fillers impart high dielectric constants to the overall composites. Due to the synergistic effect between above two materials, the energy density of the composites could be significantly improved. However, it has been found that the rise and fall of dielectric constant and breakdown strength are often opposite, which leads to limited increase in energy density. In particular, when high dielectric constant fillers are added to a high breakdown strength polymer, the breakdown strength of the composites decreases greatly, resulting in a decrease in energy density. Therefore, the preparation, modification, intermixing and mixing of polymers and fillers are particularly important [S44].

## S1.6 Electromagnetic Shielding and Microwave Absorbing Polymer Composites

With the rapid development of communication technology, electromagnetic interference (EMI) shielding materials can effectively solve the problems of electromagnetic radiation by reflecting or absorbing electromagnetic waves, and convert the electromagnetic energy into thermal energy to dissipate, becoming a hot topic in recent years [S45, S46]. Traditional EMI shielding materials are mainly based on metal and magnetic materials, which have many disadvantages of high density, easy oxidation, and mainly arouse from the reflection of electromagnetic waves, limiting its application. Due to the advantages of inherent flexibility, easy processability, ultimate scalability, chemical resistance and light weight, polymer-based EMI materials arouse great research enthusiasm. Generally, by adding fillers into polymer matrix is currently the main strategy to improve shielding performance. This filler including metals and magnetics, carbon-based materials [S47-S49]. In addition to the selection of fillers and polymer matrix, filler network morphology has important influence on the preparation of EMI shielding materials. The key factor to achieve high shielding efficiency is the formation of filler conductive network and its structural design, which would lead to different dissipation paths.

Microwave absorbing (MA) material has the ability to attenuate incident electromagnetic waves without reflection and convert the incident electromagnetic energy into heat energy or other energy dissipation within the material. The mechanism cab be divided into: dielectric loss, magnetic loss and electromagnetic impedance matching [S50]. In MA polymer

composites, filler and a portion polymer matrix can produce magnetic loss and dielectric loss. Impedance matching plays the key role in affecting the properties of MA materials. It is a synergy between dielectric loss and magnetic loss. In other words, only when there is a good impedance match between the dielectric loss and the magnetic loss can MA materials achieve excellent electromagnetic wave absorption performance. Impedance matching is affected by hybridization of filler components and morphological structure, interference, dispersion, and even filler loading. In MA polymer composites field, the processing method used is relatively simple, such as pour casting, foaming, electrospinning or hot-pressing. Using single or combined processing methods to achieve high performance MA polymer composites through morphology control is still a challenge.

## Supplementary References


[S1] X. Jia, G. Li, B. Liu, Y. Luo, G. Yang et al., Multiscale reinforcement and interfacial strengthening on epoxy-based composites by silica nanoparticle-multiwalled carbon nanotube complex. Compos. Part A Appl. Sci. Manuf. **48**, 101-109 (2013). https://doi.org/10.1016/j.compositesa.2013.01.001

[S2] H. Deng, L. Lin, M.Z. Ji, S.M. Zhang, M.B. Yang et al., Progress on the morphological control of conductive network in conductive polymer composites and the use as electroactive multifunctional materials. Prog. Polym. Sci. **39**(4), 627-655 (2014). https://doi.org/10.1016/j.progpolymsci.2013.07.007

[S3] M. Haghgoo, R. Ansari, M.K. Hassanzadeh-Aghdam, M. Nankali, Analytical formulation for electrical conductivity and percolation threshold of epoxy multiscale nanocomposites reinforced with chopped carbon fibers and wavy carbon nanotubes considering tunneling resistivity. Compos. Part A Appl. Sci. Manuf. **126**, 105616 (2019). https://doi.org/10.1016/j.compositesa.2019.105616

[S4] W. Bauhofer, J.Z. Kovacs, A review and analysis of electrical percolation in carbon nanotube polymer composites. Compos. Sci. Technol. **69**(10), 1486-1498 (2009). https://doi.org/10.1016/j.compscitech.2008.06.018

[S5] M. Jouni, A. Boudenne, G. Boiteux, V. Massardier, B. Garnier et al., Electrical and thermal properties of polyethylene/silver nanoparticle composites. Polym. Compos. **34**(5), 778-786 (2013). https://doi.org/10.1002/pc.22478

[S6] S. Wang, P. Xiao, Y. Liang, J. Zhang, Y. Huang et al., Network cracks-based wearable strain sensors for subtle and large strain detection of human motions. J. Mater. Chem. C **6**(19), 5140-5147 (2018). https://doi.org/10.1039/c8tc00433a

[S7] A.S. Kurian, V.B. Mohan, D. Bhattacharyya, Embedded large strain sensors with graphene-carbon black-silicone rubber composites. Sens. Actuator. A Phys. **282**, 206-214 (2018). https://doi.org/10.1016/j.sna.2018.09.017

[S8] S. Wang, X. Zhang, X. Wu, C. Lu, Tailoring percolating conductive networks of natural rubber composites for flexible strain sensors via a cellulose nanocrystal templated assembly. Soft Matter **12**(3), 845-852 (2016). https://doi.org/10.1039/c5sm01958c

[S9] L. Duan, D.R. D'Hooge, M. Spoerk, P. Cornillie, L. Cardon, Facile and low-cost route for sensitive stretchable sensors by controlling kinetic and thermodynamic conductive network regulating strategies. Acs Appl. Mater. Interfaces **10**(26), 22678-22691 (2018).

https://doi.org/10.1021/acsami.8b03967

[S10] Y.A. Samad, Y. Li, S.M. Alhassan, K. Liao, Novel graphene foam composite with adjustable sensitivity for sensor applications. ACS Appl. Mater. Interfaces **7**(17), 9195-9202 (2015). https://doi.org/10.1021/acsami.5b01608

[S11] T.Q. Trung, N.E. Lee, Recent progress on stretchable electronic devices with intrinsically stretchable components. Adv. Mater. **29**(3), 1603167 (2017). https://doi.org/10.1002/adma.201603167

[S12] G. Li, K. Dai, M. Ren, Y. Wang, G. Zheng et al., Aligned flexible conductive fibrous networks for highly sensitive, ultrastretchable and wearable strain sensors. J. Mater. Chem. C **6**(24), 6575-6583 (2018). https://doi.org/10.1039/c8tc01924j

[S13] X. Ye, Z. Yuan, H. Tai, W. Li, X. Du et al., A wearable and highly sensitive strain sensor based on a polyethylenimine-rGO layered nanocomposite thin film. J. Mater. Chem. C **5**(31), 7746-7752 (2017). https://doi.org/10.1039/c7tc01872j

[S14] Q. Liu, J. Chen, Y. Li, G. Shi, High-performance strain sensors with fish-scale-like graphene-sensing layers for full-range detection of human motions. ACS Nano **10**(8), 7901-7906 (2016). https://doi.org/10.1021/acsnano.6b03813

[S15] B. Park, J. Kim, D. Kang, C. Jeong, K.S. Kim et al., Dramatically enhanced mechanosensitivity and signal-to-noise ratio of nanoscale crack-based sensors: effect of crack depth. Adv. Mater. **28**(37), 8130-8137 (2016). https://doi.org/10.1002/adma.201602425

[S16] D. Kang, P.V. Pikhitsa, Y.W. Choi, C. Lee, S.S. Shin et al., Ultrasensitive mechanical crack-based sensor inspired by the spider sensory system. Nature **516**(7530), 222-226 (2014). https://doi.org/10.1038/nature14002

[S17] Y. Yang, X. Huang, Z. Cao, G. Chen, Thermally conductive separator with hierarchical nano/microstructures for improving thermal management of batteries. Nano Energy **22**, 301-309 (2016). https://doi.org/10.1016/j.nanoen.2016.01.026

[S18] X. Huang, P. Jiang, T. Tanaka, A review of dielectric polymer composites with high thermal conductivity. IEEE Electr. Insul. M. **27**(4), 8-16 (2011). https://doi.org/10.1109/mei.2011.5954064

[S19] N. Burger, A. Laachachi, M. Ferriol, M. Lutz, V. Toniazzo et al., Review of thermal conductivity in composites: mechanisms, parameters and theory. Prog. Polym. Sci. **61**, 1-28 (2016). https://doi.org/10.1016/j.progpolymsci.2016.05.001

[S20] W. Zhao, J. Kong, H. Liu, Q. Zhuang, J. Gu et al., Ultra-high thermally conductive and rapid heat responsive poly(benzobisoxazole) nanocomposites with self-aligned graphene. Nanoscale **8**(48), 19984-19993 (2016). https://doi.org/10.1039/c6nr06622d

[S21] A.M. Marconnett, N. Yamamoto, M.A. Panzer, B.L. Wardle, K.E. Goodson, Thermal conduction in aligned carbon nanotube-polymer nanocomposites with high packing density. ACS Nano **5**(6), 4818-4825 (2011). https://doi.org/10.1021/nn200847u

[S22] F. Kargar, Z. Barani, M. Balinskiy, A.S. Magana, J.S. Lewis et al., Dual-functional

graphene composites for electromagnetic shielding and thermal management. Adv. Electron. Mater. **5**(1), 1800558 (2019). https://doi.org/10.1002/aelm.201800558

[S23] Q. Li, Z. Xue, J. Zhao, C. Ao, X. Jia et al., Mass production of high thermal conductive boron nitride/nanofibrillated cellulose composite membranes. Chem. Eng. J. **383**, 123101 (2020). https://doi.org/10.1016/j.cej.2019.123101

[S24] Y. Yao, X. Zeng, G. Pan, J. Sun, J. Hu et al., Interfacial engineering of silicon carbide nanowire/cellulose microcrystal paper toward high thermal conductivity. ACS Appl. Mater. Interfaces **8**(45), 31248-31255 (2016). https://doi.org/10.1021/acsami.6b10935

[S25] M. Hu, J. Feng, K.M. Ng, Thermally conductive PP/AlN composites with a 3D segregated structure. Comp. Sci. Technol. **110**, 26-34 (2015). https://doi.org/10.1016/j.compscitech.2015.01.019

[S26] G.H. Kim, D. Lee, A. Shanker, L. Shao, M.S. Kwon et al., High thermal conductivity in amorphous polymer blends by engineered interchain interactions. Nat. Mater. **14**(3), 295-300 (2015). https://doi.org/10.1038/nmat4141

[S27] H. Chen, V.V. Ginzburg, J. Yang, Y. Yang, W. Liu et al., Thermal conductivity of polymer-based composites: fundamentals and applications. Prog. Polym. Sci. **59**, 41-85 (2016). https://doi.org/10.1016/j.progpolymsci.2016.03.001

[S28] G. Chen, W. Xu, D. Zhu, Recent advances in organic polymer thermoelectric composites. J. Mater. Chem. C **5**(18), 4350-4360 (2017). https://doi.org/10.1039/c6tc05488a

[S29] J. Cao, J.D. Querales-Flores, S. Fahy, I. Savić, Thermally induced band gap increase and high thermoelectric figure of merit of n-type PbTe. Mater. Today Phys. **12**, 100172 (2020). https://doi.org/10.1016/j.mtphys.2019.100172

[S30] H. Alam, S. Ramakrishna, A review on the enhancement of figure of merit from bulk to nano-thermoelectric materials. Nano Energy **2**(2), 190-212 (2013). https://doi.org/10.1016/j.nanoen.2012.10.005

[S31] Q. Zhang, Y. Sun, W. Xu, D. Zhu, Organic thermoelectric materials: emerging green energy materials converting heat to electricity directly and efficiently. Adv. Mater. **26**(40), 6829-6851 (2014). https://doi.org/10.1002/adma.201305371

[S32] M. He, F. Qiu, Z. Lin, Towards high-performance polymer-based thermoelectric materials. Energy Environ. Sci. **6**(5), 1352-1361 (2013). https://doi.org/10.1039/c3ee24193a

[S33] X. Hu, G. Chen, X. Wang, H. Wang, Tuning thermoelectric performance by nanostructure evolution of a conducting polymer. J. Mater. Chem. A **3**(42), 20896-20902 (2015). https://doi.org/10.1039/c5ta07381b

[S34] D.M. Rowe, G. Min, Evaluation of thermoelectric modules for power generation. J. Power Sources **73**(2), 193-198 (1998). https://doi.org/10.1016/s0378-7753(97)02801-2

[S35] Q. Zhang, Y. Sun, W. Xu, D. Zhu, Thermoelectric energy from flexible P3HT films doped with a ferric salt of triflimide anions. Energy Environ. Sci. **5**(11), 9639-9644

(2012). https://doi.org/10.1039/c2ee23006b

[S36] G.H. Kim, L. Shao, K. Zhang, K.P. Pipe, Engineered doping of organic semiconductors for enhanced thermoelectric efficiency. Nat. Mater. **12**(8), 719-723 (2013). https://doi.org/10.1038/nmat3635

[S37] S. Garreau, G. Louarn, J.P. Buisson, G. Froyer, S. Lefrant, In situ spectroelectrochemical Raman studies of poly(3,4-ethylenedioxythiophene) (PEDT). Macromolecules **32**(20), 6807-6812 (1999). https://doi.org/10.1021/ma9905674

[S38] H. Jin, J. Li, J. Iocozzia, X. Zeng, P.C. Wei et al., Hybrid organic-inorganic thermoelectric materials and devices. Angew. Chem. Int. Ed. **58**(43), 15206-15226 (2019). https://doi.org/10.1002/anie.201901106

[S39] C. Yu, K. Choi, L. Yin, J.C. Grunlan, Light-weight flexible carbon nanotube based organic composites with large thermoelectric power factors. ACS Nano **5**(10), 7885-7892 (2011). https://doi.org/10.1021/nn202868a

[S40] T.D. Huan, S. Boggs, G. Teyssedre, C. Laurent, M. Cakmak et al., Advanced polymeric dielectrics for high energy density applications. Prog. Mater. Sci. **83**, 236-269 (2016). https://doi.org/10.1016/j.pmatsci.2016.05.001

[S41] X. Huang, P. Jiang. Core-shell structured high-*k* polymer nanocomposites for energy storage and dielectric applications. Adv. Mater. **27**(3), 546-554 (2015). https://doi.org/10.1002/adma.201401310

[S42] W. Xia, Z. Zhang, PVDF-based dielectric polymers and their applications in electronic materials. IET Nanodielectrics **1**(1), 17-31 (2018). https://doi.org/10.1049/iet-nde.2018.0001

[S43] M. Ritamäki, I. Rytöluoto, K. Lahti, High temperature and ageing test methods to characterize the dielectric properties of BOPP capacitor films. 2017 IEEE Conference on Electrical Insulation and Dielectric Phenomenon (CEIDP), Fort Worth, TX, USA. 266-269 (2017). https://doi.org/10.1109/CEIDP.2017.8257518

[S44] X. Zhang, C. Tan, Y. Ma, F. Wang, W. Yang, $BaTiO_3$ @carbon/silicon carbide/ poly(vinylidene fluoride-hexafluoropropylene) three-component nanocomposites with high dielectric constant and high thermal conductivity. Compos. Sci. Technol. **162**, 180-187 (2018). https://doi.org/10.1016/j.compscitech.2018.05.001

[S45] M.S. Cao, Y.Z. Cai, P. He, J.C. Shu, W.Q. Cao et al., 2D MXenes: electromagnetic property for microwave absorption and electromagnetic interference shielding. Chem. Eng. J. **359**, 1265-1302 (2019). https://doi.org/10.1016/j.cej.2018.11.051

[S46] P. Kumar, U.N. Maiti, A. Sikdar, T.K. Das, A. Kumar et al., Recent advances in polymer and polymer composites for electromagnetic interference shielding: Review and future prospects. Polym. Rev. **59**(4), 687-738 (2019). https://doi.org/10.1080/15583724.2019.1625058

[S47] R. Bian, G. He, W. Zhi, S. Xiang, T. Wang et al., Ultralight MXene-based aerogels with high electromagnetic interference shielding performance. J. Mater. Chem. C **7**(3), 474-478 (2019). https://doi.org/10.1039/c8tc04795b

[S48] H. Xu, X. Yin, X. Li, M. Li, S. Liang et al., Lightweight $Ti_2CTx$ MXene/poly(vinyl alcohol) composite foams for electromagnetic wave shielding with absorption-dominated feature. ACS Appl. Mater. Interfaces **11**(10), 10198-10207 (2019). https://doi.org/10.1021/acsami.8b21671

[S49] Y. Bhattacharjee, I. Arief, S. Bose, Recent trends in multi-layered architectures towards screening electromagnetic radiation: challenges and perspectives. J. Mater. Chem. C **5**(30), 7390-7403 (2017). https://doi.org/10.1039/c7tc02172k

[S50] B. Quan, X. Liang, G. Ji, J. Ma, P. Ouyang et al., Strong electromagnetic wave response derived from the construction of dielectric/magnetic media heterostructure and multiple interfaces. ACS Appl. Mater. Interfaces **9**(11), 9964-9974 (2017). https://doi.org/10.1021/acsami.6b15788
